# Overexpression of the essential Sis1 chaperone reduces TDP-43 effects on toxicity and proteolysis

**DOI:** 10.1371/journal.pgen.1006805

**Published:** 2017-05-22

**Authors:** Sei-Kyoung Park, Joo Y. Hong, Fatih Arslan, Vydehi Kanneganti, Basant Patel, Alex Tietsort, Elizabeth M. H. Tank, Xingli Li, Sami J. Barmada, Susan W. Liebman

**Affiliations:** 1 Department of Pharmacology, University of Nevada, Reno, Nevada, United States of America; 2 Department of Biological Sciences, University of Illinois at Chicago, Chicago, Illinois, United States of America; 3 Department of Biochemistry and Molecular Biology, University of Nevada, Reno, Nevada, United States of America; 4 Department of Neurology, University of Michigan, Ann Arbor, Michigan, United States of America; The University of Arizona, UNITED STATES

## Abstract

Amyotrophic lateral sclerosis (ALS) is a devastating neurodegenerative disease characterized by selective loss of motor neurons with inclusions frequently containing the RNA/DNA binding protein TDP-43. Using a yeast model of ALS exhibiting TDP-43 dependent toxicity, we now show that TDP-43 overexpression dramatically alters cell shape and reduces ubiquitin dependent proteolysis of a reporter construct. Furthermore, we show that an excess of the Hsp40 chaperone, Sis1, reduced TDP-43’s effect on toxicity, cell shape and proteolysis. The strength of these effects was influenced by the presence of the endogenous yeast prion, [*PIN*^+^]. Although overexpression of Sis1 altered the TDP-43 aggregation pattern, we did not detect physical association of Sis1 with TDP-43, suggesting the possibility of indirect effects on TDP-43 aggregation. Furthermore, overexpression of the mammalian Sis1 homologue, DNAJB1, relieves TDP-43 mediated toxicity in primary rodent cortical neurons, suggesting that Sis1 and its homologues may have neuroprotective effects in ALS.

## Author summary

Many neurodegenerative diseases are associated with aggregation of specific proteins. Thus we are interested in factors that influence the aggregation and how the aggregated proteins are associated with pathology. Here, we study a protein called TDP-43 that is frequently aggregated in the neurons of patients with amyotrophic lateral sclerosis (ALS). TDP-43 aggregates and is toxic when expressed in yeast, providing a useful model for ALS. Remarkably, a protein that modified TDP-43 toxicity in yeast successfully predicted a new ALS susceptibility gene in humans. We now report a new modifier of TDP-43 toxicity, Sis1. We show that expression of TDP-43 in yeast inhibits degradation of damaged protein, while overexpression of Sis1 restores degradation. Thus suggests a link between protein degradation and TDP-43 toxicity. Furthermore we show that a mammalian protein similar to Sis1 reduces TDP-43 toxicity in primary rodent neurons. This identifies the mammalian Sis1-like gene as a new ALS therapeutic target and possible susceptibility gene.

## Introduction

Amyotrophic lateral sclerosis (ALS), often referred to as "Lou Gehrig's Disease", is a progressive neurodegenerative disorder characterized by degeneration and ultimately death of motor neurons in the brain and the spinal cord [1,2]. The most common pathologic characteristic of ALS is the formation of cytoplasmic inclusions rich in the transactive response element DNA/RNA binding protein of 43 kDa (TDP-43) [3–7]. Furthermore, mutations in the gene encoding TDP-43 (*TARDBP*) cause familial ALS [8–11]. Cytoplasmic TDP-43 inclusions are also common in the majority of individuals with frontotemporal dementia (FTD) [3,4], suggesting that ALS and FTD are linked by a common disease mechanism involving TDP-43 mislocalization and aggregation.

TDP-43 is an RNA/DNA-binding protein [12,13] that is found in the nucleus under normal conditions [3,4], and is associated with cytoplasmic stress granules during stress [14]. TDP-43 harbors two RNA Recognition Motif (RRM) domains and a C-terminal glycine-rich region, where most of the ALS-linked mutations are found [12,13,15]. An algorithm to identify human proteins with aggregation prone, prion-like domains ranked TDP-43 69^th^ among the entire human proteome, because of its C-terminus (residues 277–414) ([16] also see [17–19]). The abnormal localization of wild-type or mutant versions of this protein to cytoplasmic foci in the absence of stress is associated with ALS [3,5,6,20].

Prions are self-seeding conformational variants of particular proteins [21]. The conversion of largely α-helical cellular prion protein PrP^C^ into self-seeding fibrous β-sheet-rich ordered aggregates (amyloids) called PrP^Sc^ (associated with scrapie) is the causative agent of prion diseases in mammals [22]. A conformational change of a disease-specific soluble cellular protein to an aggregated form that seeds further aggregation was also shown for Alzheimer’s disease, Parkinson’s disease and ALS [17,18,23–25].

In addition, several yeast proteins have been shown to convert from soluble to self-seeding amyloid conformations called prions, and are associated with transmissible phenotypes in yeast [21,26–33]. Interestingly, yeast prions can enhance the rate of *de novo* aggregation of heterologous prion proteins, presumably by a cross-seeding mechanism. Indeed, the endogenous yeast prion, [*PIN*^*+*^] [29,34], is required for the aggregation and toxicity of human Huntington's disease Htt protein with an expanded polyglutamine (polyQ) tract when expressed in yeast [35,36].

Chaperones, including Sis1, an Hsp40 chaperone, are required for the propagation of yeast prions [37–42]. In addition to its high nuclear concentration, Sis1 is also diffusely localized in the cytoplasm in exponentially growing cells [43]. However, when prion or other amyloid cytoplasmic aggregates are present, Sis1 also co-localizes with them creating cytoplasmic Sis1 foci [44,45]. Sis1 is required for the fragmentation of prion oligomers. Indeed, reduced or overexpressed levels of Sis1 lead to larger or smaller prion oligomers [37–41]. The new aggregate ends created by this fragmentation are required for prion propagation. Furthermore, the smaller aggregates created by fragmentation are more easily transmitted to daughter cells, also promoting prion propagation [45–47].

Overexpression of Sis1 rescues yeast from toxicity associated with overexpression of several prion or prion-like proteins. For example, overexpression of Sis1 reduces [*PIN*^*+*^] dependent polyQ aggregation and toxicity [48]. The mechanism of rescue is in part because the polyQ aggregates sequester Sis1, inhibiting it from its normal role of transporting ubiquitinated misfolded proteins in the cytoplasm to the nucleus for proteasomal degradation [44].

Similarly, overexpression of Sis1 rescues cells from [*PIN*^+^] dependent toxicity of an overexpressed prion-like protein Pin4C. When overexpressed in the presence of [*PIN*^+^], Pin4C formed large hyperphosphorylated aggregates that co-localized with Sis1 and reduced degradation of a ubiquitin proteasome system (UPS) reporter protein. Aggregation, hyperphosphorylation and toxicity were suppressed by overexpression of Sis1 [45,49].

Overexpression of Sis1 also rescues [*PIN*^+^] dependent toxicity caused by overexpression of the [*PIN*^+^] prion protein, Rnq1. Here, overexpressed Sis1 appears to enhance aggregation of toxic detergent soluble Rnq1 oligomers into apparently non-toxic larger detergent resistant aggregates in the nucleus [50,51].

Yeast is a useful organism to study the conversion of soluble disease-specific protein into amyloid or prion-like aggregates [35,48,52–56]. Overexpressed human TDP-43 forms cytoplasmic aggregates in yeast associated with toxicity [53]. Both wild-type TDP-43, which accumulates in the majority of sporadic ALS, and mutant TDP-43, associated with familial ALS, elicit cell death when overexpressed. However, at near-endogenous expression levels mutant TDP-43 is significantly more toxic than wild-type TDP-43 [57] consistent with the near complete penetrance of *TARDBP* mutations in familial ALS [8].

Unlike most other prion-like aggregating proteins, TDP-43 aggregates do not appear to be typical amyloids [58]. Unbiased screens for overexpression or deletion modifiers of TDP-43 toxicity identified numerous yeast proteins as candidates for involvement in the TDP-43 toxicity cascade. The identification of one such modifier, Pbp1, with a human homologue *ATXN2*, led to the discovery that the length of polyglutamine expansions in *ATXN2* is associated with increased risk for ALS [59]. This clearly established the power of the yeast model in understanding human disease.

Here, we identify a new modifier by showing that excess Sis1 reduces the toxicity of overexpressed TDP-43. Likewise, overexpression of the mammalian Sis1 homologue, DNAJB1, reduces TDP-43-mediated toxicity in primary rodent cortical neurons, suggesting that Sis1 and its homologues may have neuroprotective effects in ALS. Finally, we provide evidence that TDP-43 impedes the UPS-mediated degradation of cytosolic misfolded proteins and that overexpression of Sis1 restores degradation even in the presence of excess TDP-43.

## Results

### Overexpression of TDP-43 causes altered cell morphology

**Fig 1:**
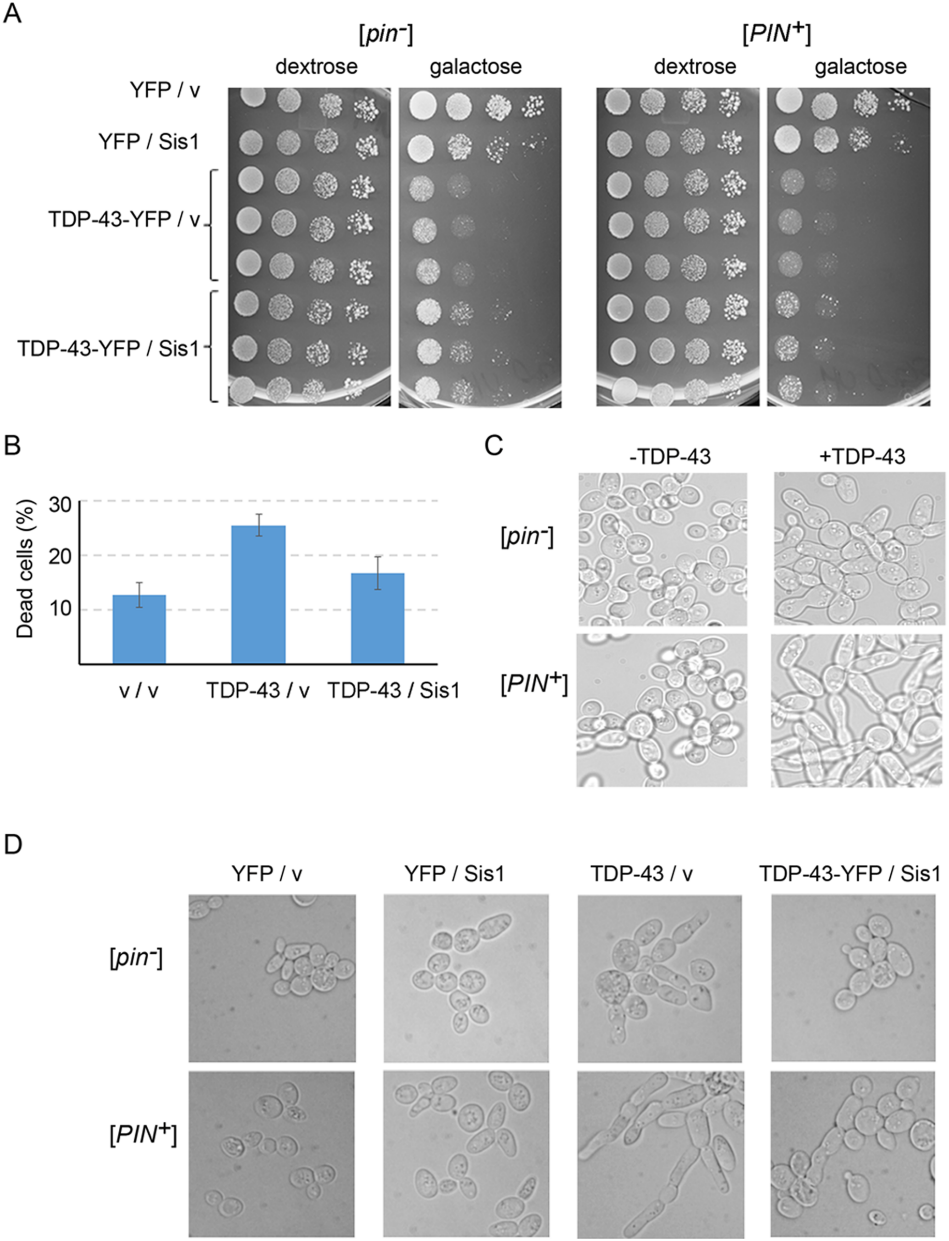
Overexpression of TDP-43 causes growth inhibition and altered cell morphology, both of which are partially relieved by overexpression of the Sis1 Hsp40 chaperone. (A) TDP-43 growth inhibition is more extreme in [*PIN*^+^] 74D-694 cells and is partially relieved by Sis1 overexpression. [*PIN*^*+*^] (L1749) and [*pin*^*-*^] (L2910) cells transformed with p*GAL1-TDP-43-YFP* (p2042), or p*YFP* control (p1752) and p*GAL1-SIS1* (p1767), or empty control (p1768) plasmids, were selected on SD-Ura-Trp plates. Normalized suspensions of cells taken from SD-Ura-Trp were 10X serially diluted in water and 15 μl were spotted on SD-Ura-Trp (dextrose), and 2% Gal-Ura-Trp (galactose) plates, which were photographed after 3 (dextrose) or 5 (galactose) days of incubation at 30°C. (B) Sis1 overexpression partially rescues cells from death caused by TDP-43 overexpression. Viability of [*pin*^-^] cells transformed with p*GAL1-TDP-43* (p2195), or control (p2186) and p*GAL1*-*SIS1* (p1759), or control (p484) plasmids and grown for 72 h on 2% raffinose + 2% galactose selective media was determined by staining dead cells with trypan blue. Shown is the average and standard error from three separate experiments. (C) TDP-43 overexpression causes cell elongation, which is more extreme in [*PIN*^+^] cells. Cells transformed with p*GAL1-TDP-43* (p2055) or control (p484) were grown overnight in liquid SRaf-Ura, and galactose was added to induce TDP-43 expression for 24 h prior to imaging. (D) Sis1 overexpression partially relieves cell elongation caused by TDP-43-YFP overexpression both in [*pin*^-^] and [*PIN*^+^] cells. Cells co-transformed with p*GAL1-TDP-43-YFP* (p2042), or control (p1752) and p*GAL1-SIS1* (p1767), or control (p1768) plasmids on SD-Ura-Trp were grown overnight in liquid SRaf-Ura-Trp to OD600: 0.5 to 0.7. Galactose was added to 2% to induce TDP-43-YFP and cells were imaged following fixation at 24 h.

Although overexpressed polyQ or Pin4C only form large aggregates and causes toxicity in [*PIN*^+^] cells [35,49], overexpressed TDP-43 does so in both [*PIN*^+^] and [*pin*^-^] yeast [53,60]. Likewise, we saw that overexpressed TDP-43 inhibited growth in both [*PIN*^+^] and [*pin*^-^] cells (Fig 1A and S1 Fig). Although we noted some variability between transformants and strain backgrounds, there was often a slightly stronger inhibition in [*PIN*^+^] vs. isogenic [*pin*^-^] cells (Fig 1A).

**Fig 2:**
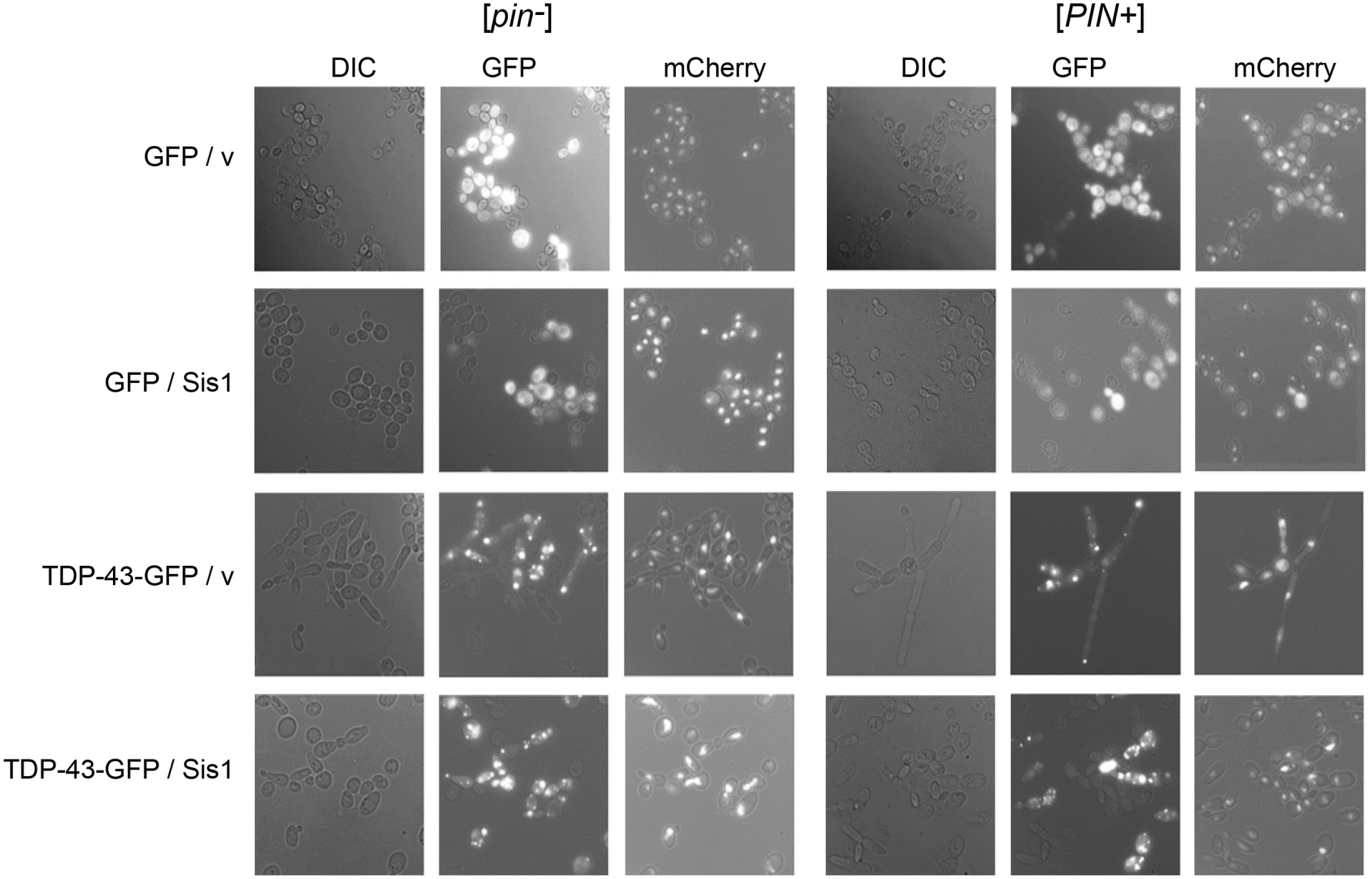
Cells elongated due to TDP-43-GFP expression retain single nuclei. [*PIN*^+^] (L3491) and [*pin*^-^] (L3496) cells with nuclei marked with *HTB1-mCherry* were co-transformed with controls p*GAL1-GFP* (p1764) and p*GAL1* empty vector (p1768) “GFP/ v”, control and p*GAL1-Sis1* (p1767) “GFP/ Sis1”, p*GAL1-TDP-43-GFP* (p2288) and control “TDP-43-GFP/ v” and “TDP-43-GFP/ Sis1”. Transformants were cultured in SD-Ura-Trp medium to OD600 ≤ 0.5 and re-inoculated into SGal/Raf-Ura-Trp containing 0.5% raffinose and 2% galactose to induce TDP-43-GFP and Sis1. Cells were fixed at 24 h and imaged.

In addition, we found that overexpressed TDP-43 altered cell shape, and again the effect was slightly but reproducibly stronger in [*PIN*^+^] cells. We examined this effect in three strain backgrounds for cells grown on 2% galactose plates for 4 days (S2 Fig). In strain 74D-694 [*PIN*^+^] nearly every cell showed altered morphology, with less extreme numbers and less elongation in [*pin*^-^] cells. Elongated cells were also easily seen in BY4741 and W303 strain backgrounds although especially in BY4741 many cells did not have an altered shape. Likewise, 74D-694 overexpressing TDP-43 for 24 h in liquid were elongated and bloated relative to control cells without TDP-43 overexpression (Fig 1C and 1D). Using cells with the endogenous nuclear Htb1 protein tagged with mCherry [61], we could see that the bloated/elongated cells each have only one non-fragmented nucleus and so did not result from a failure of cytokinesis (Fig 2). Finally, the cell morphological change was proportional to the TDP-43 expression level (S3 Fig).

### Overexpression of the Hsp40 chaperone, Sis1, relieves effects of TDP-43 on cell toxicity and shape

Since overexpression of Sis1 suppressed toxicity of other overexpressed aggregating proteins (polyQ, Rnq1 and Pin4C) [44,49–51] we decided to look at the effect of Sis1 on TDP-43 toxicity even though previous screens for modifiers of TDP-43 and FUS toxicity in yeast did not uncover Sis1 [54,59,62].

We compared growth of cells overexpressing TDP-43-YFP with those co-overexpressing Sis1 and TDP-43-YFP. Excess Sis1 reduced growth inhibition due to TDP-43 in both [*pin*^*-*^] and [*PIN*^*+*^] cells in three strain backgrounds. The rescue appeared to be weaker in 74D-694 [*PIN*^+^] cells where TDP-43 is more toxic (Fig 1A). While overexpression of Sis1 did not completely suppress TDP-43 toxicity, it altered TDP-43 toxicity more efficiently than previously reported modifiers of TDP-43 toxicity: overexpression of Hsp104, Pbp1 or human Upf1 [59,63–65] (S1 Fig).

**Fig 3:**
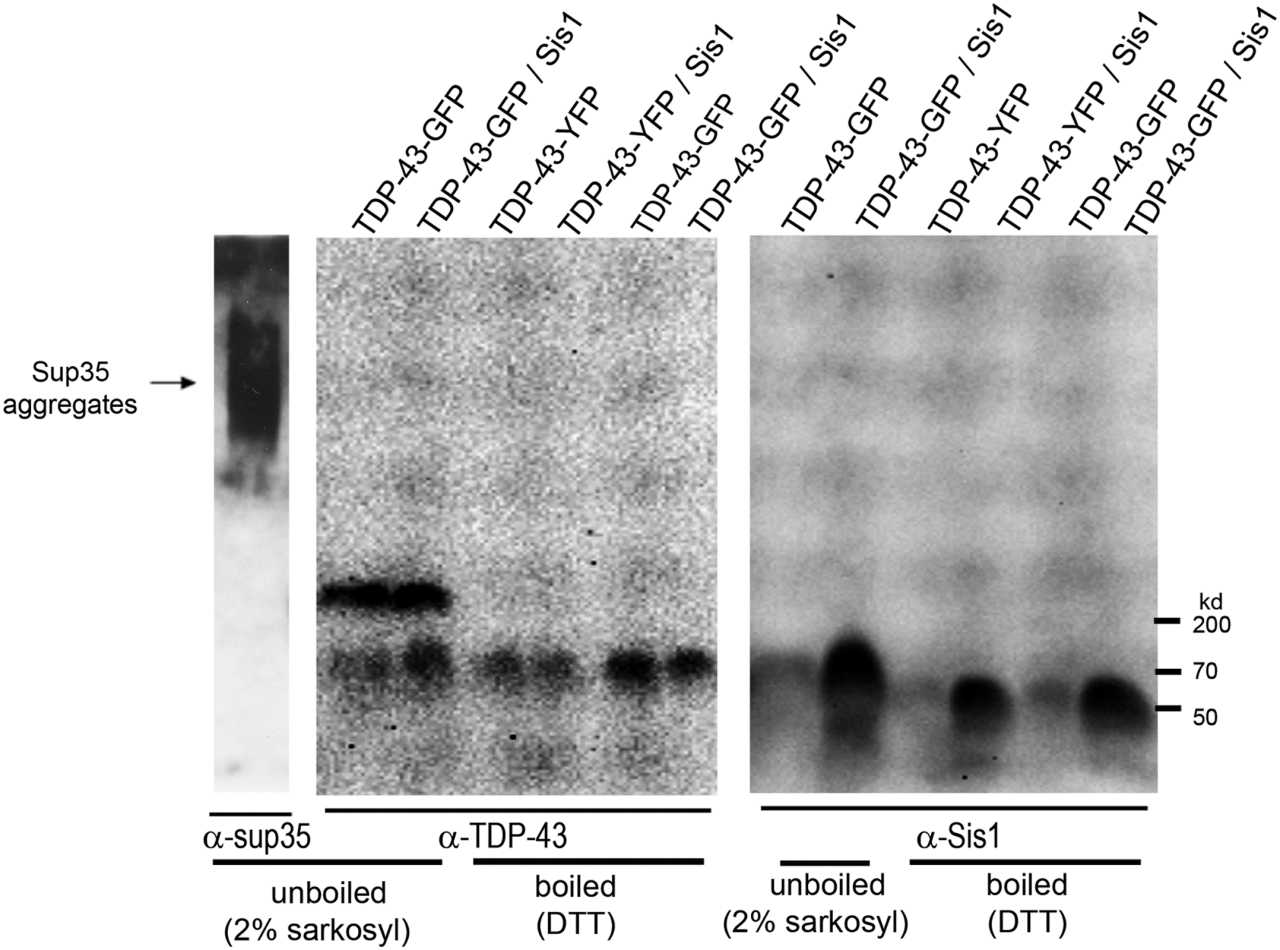
TDP-43 aggregates form sarkosyl-resistant oligomers that are not affected by Sis1 overexpression. TDP-43 aggregates form sarkosyl-resistant oligomers. [*PIN*^+^] cells (L1749) expressing *GAL1-*regulated TDP-43-YFP (p2042) or TDP-43-GFP (p2288) and *GAL1*-regulated *SIS1* (p1767) or empty control (p1768) plasmids, were grown on 2% Gal for 24 h. Proteins were then extracted and treated with 2% sarkosyl for 7 minutes (unboiled) or, to visualized monomers, were boiled for 5 minutes with 2% SDS with DTT (boiled). Proteins were resolved in a 1.5% agarose semi-denaturing gel (SDD-AGE) and visualized with an anti-TDP-43 antibody. As a positive control, [*PSI*^+^] oligomers were also visualized using an anti-Sup35C antibody (BE4, mouse monoclonal against Sup35C). After TDP-43-GFP detection, the membrane was stripped and reprobed with Sis1 antibody (kind gift of E. Craig). Protein size markers were from BioRad. TDP-43-GFP or -YFP and Sis monomer are respectively ~70 and 39.5 kDa. In unboiled samples, Sis1 runs as a dimer.

Overexpression of Sis1 also reduced cell death (Fig 1B) and the level of cell bloating/elongation associated with TDP-43 overexpression (Figs 1D and 2). Sis1 overexpression did not cause these effects by reducing the level of TDP-43 or by curing cells of [*PIN*^+^], because TDP-43 levels remained unchanged (Fig 3) and Sis1 overexpression does not cure cells of [*PIN*^+^] [66] (S4 Fig).

### TDP-43 overexpression inhibits the degradation of a UPS reporter protein and overexpression of Sis1 reverses this effect

**Fig 4:**
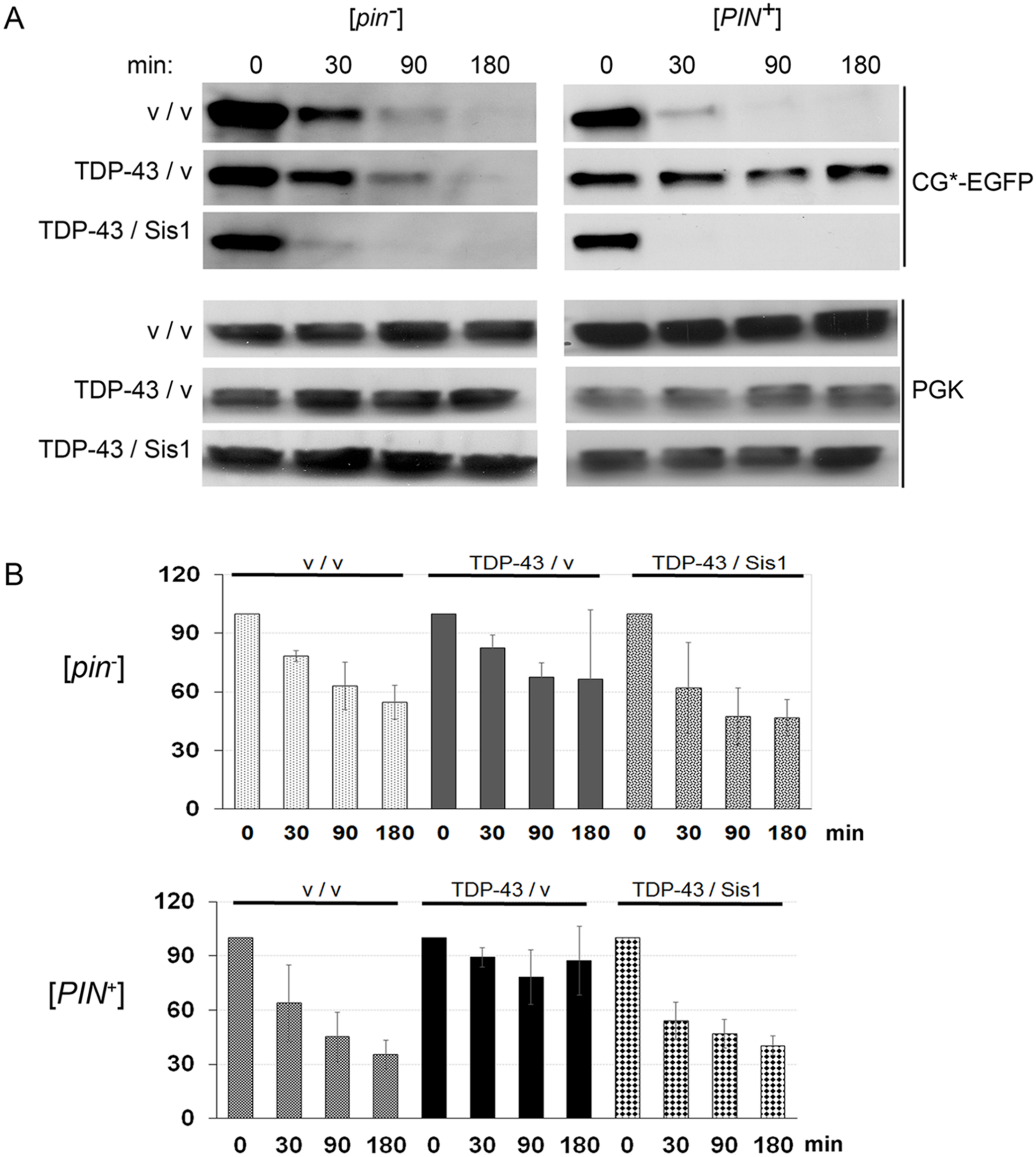
TDP-43 overexpression inhibits degradation of a UPS-reporter and co-expression of Sis1 restores that degradation. Degradation of CG*, the mutant version of the secretory protein carboxypeptidase Y lacking its signal sequence tagged with EGFP (ΔssCPY*) was used to report functionality of the ubiquitin proteasome system (UPS). Levels of CG* were determined in cell lysates [103] harvested at 0, 30, 90 and 180 minutes following inhibition of new protein synthesis with cycloheximide. [*pin*^-^] (L2910) or [*PIN*^+^] (L1749) cells expressing *GAL1* regulated CG*-EGFP (p2154) and either control empty vectors (p2039, p1768) “v / v”; TDP-43 (p2055) and control “TDP-43 / v”; or TDP-43 and Sis1 (p1767) “TDP-43 / Sis1”, were grown in liquid SR-Trp-Ura-His to OD 600 ≥ 0.7. Galactose to 2% was added and cells were grown for 24 h to induce TDP-43, Sis1 and CG* expression. Protein synthesis was then inhibited with 0.75 ([*pin*^-^]) or 0.5 ([*PIN*^+^]) μg/ml cycloheximide for the times indicated. (A) A representative Western blot. Normalized cell lysates were run on SDS-PAGE followed by immunoblotting with anti-GFP (1:5,000, Roche) for CG*-EGFP level and anti-Pgk1 (1:10,000, Novex) as an internal loading control. (B) Quantification of three blots showing normalized ratio of CG*-EGFP and Pgk1. Standard error for three blots is shown.

Previous results showed that overexpression of polyQ or Pin4C in [*PIN*^+^] cells reduced degradation of a UPS reporter, CG* [44,49], and that the overexpression of Sis1 reversed this polyQ effect [44,49]. Thus we asked if TDP-43 overexpression likewise affects clearance of CG*. CG* is the mutant version of the secretory protein carboxypeptidase Y lacking its signal sequence and tagged with GFP (ΔssCPY*). Normally, CG* is rapidly degraded via the UPS, dependent on Hsp40 and Hsp70 chaperones [44]. After inhibition of new protein synthesis with cycloheximide (CHX), CG* degradation was inhibited by TDP-43 overexpression (Fig 4). The effect was seen in both [*pin*^*-*^] and [*PIN*^*+*^] 74D-694 cells but was more dramatic in [*PIN*^*+*^] cells where TDP-43 is more toxic. Furthermore, simultaneous overexpression of Sis1 and TDP-43 resulted in rapid degradation of CG*. This is consistent with the hypothesis that TDP-43 toxicity in part results from interference with the UPS system.

Since deletion of *HSP104* prevents propagation of several yeast prions [21] we looked for effects of *hsp104Δ* on the ability of Sis1 overexpression to reduce TDP-43 toxicity. No effects were detected when comparing cells with wild-type *HSP104* vs. *hsp104Δ* (S5A Fig). Also, as Sis1 is an Ssa1 co-chaperone [67,68] we looked for effects of lowered Ssa1 activity on the ability of Sis1 overexpression to reduce TDP-43 toxicity. This experiment was complicated by the fact that in addition to Sis1 being a co-chaperone for Ssa1, it is presumed to be a co-chaperone of the other members of the Ssa family (Ssa2, Ssa3 and Ssa4). Furthermore, deletion of all members of this family is lethal and deletion of one member is compensated for by alteraed expression of other members. Thus, we used the dominant negative *SSA1-21* mutant in an *ssa2Δ* background which has a dramatic effect on Ssa family activity causing loss of the [*PSI*^+^] prion [69]. We found that this alteration in Ssa activity did not detectably affect the ability of Sis1 overexpression to reduce TDP-43 toxicity (S5B Fig).

### TDP-43 forms sarkosyl resistant aggregates that are not affected by Sis1 overexpression

TDP-43 aggregates are soluble in 2% SDS at room temperature and to fail to stain with the amyloid dye thioflavin T [53]. We asked if the TDP-43 aggregates had the amyloid-like property of being insoluble in a different detergent, sarkosyl, because some pathological TDP-43 aggregates were resistant to sarkosyl [3,4] and because *in vitro* fibrillized TDP-43 remains insoluble in sarkosyl [70]. Indeed, when we treated lysates of cells harboring TDP-43 aggregates with sarkosyl at room temperature (Fig 3), TDP-43-YFP oligomers were visible in unboiled samples. The size and abundance of these oligomers was unchanged by Sis1 overexpression and Sis1 was not detected in these oligomers.

### Overexpression of Sis1 alters, but does not inhibit TDP-43 aggregation

**Fig 5:**
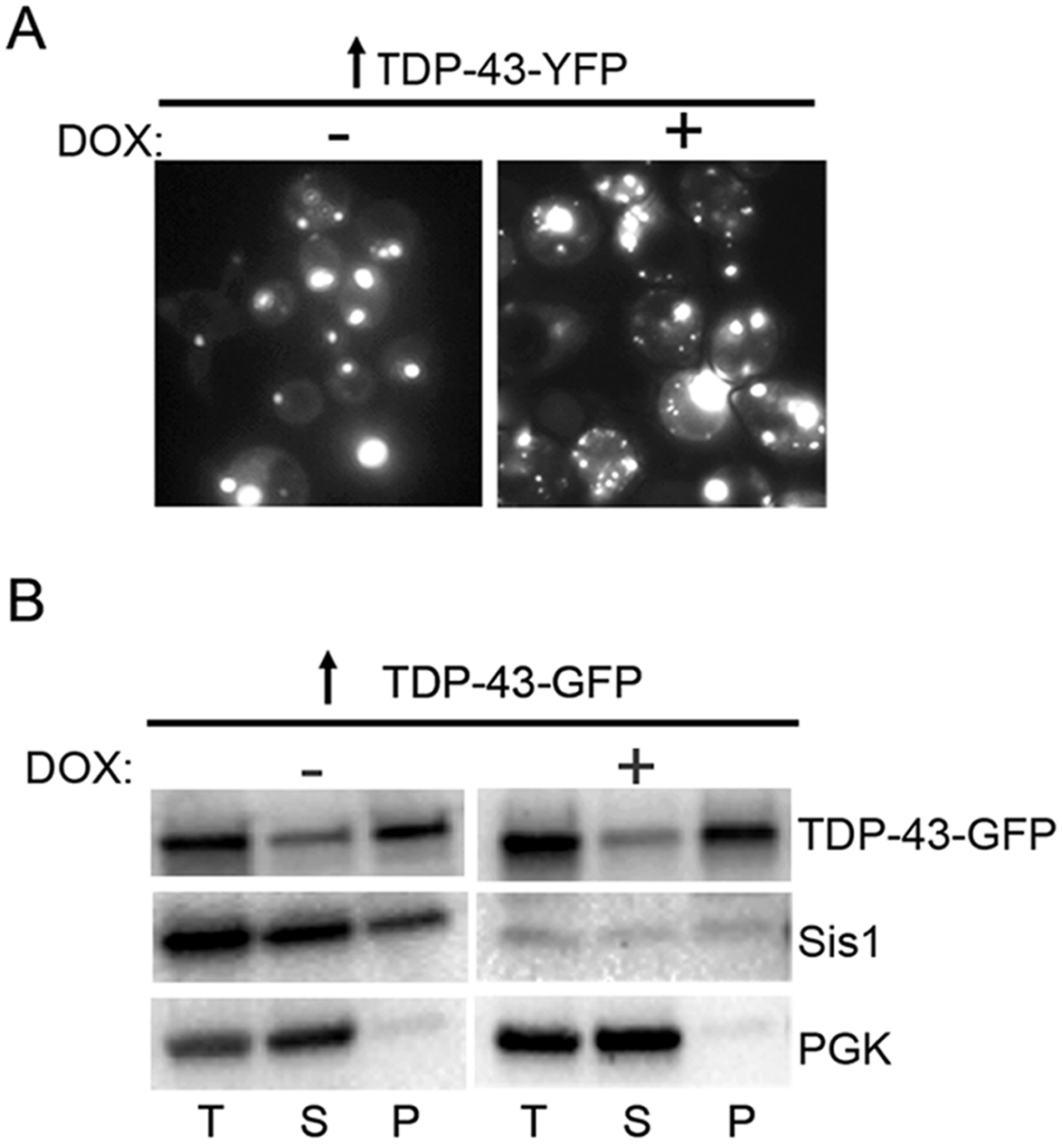
Depletion of Sis1 affects TDP-43 aggregation without causing a significant difference in TDP-43 soluble vs. aggregated distribution. W303 [*PIN*^+^] cells with a deletion of *SIS1* and carrying pCM184-*TET*^*R*^*-SIS1* (Y2182) transformed with *GAL1* regulated YFP (p2042) or GFP tagged (p2288) TDP-43, were grown on plasmid selective 2% dextrose plates. (A) Lowering Sis1 expression level alters the TDP43-YFP aggregation pattern. Transformants inoculated into 2% raffinose + 2% galactose liquid selective medium were immediately aliquoted into two tubes. Doxycycline (10 μg/ml) was added to only one such tube to inhibit Sis1 expression (DOX +). Cells without doxycycline (DOX -) had overexpressed Sis1 levels. After 24 h, both cultures had big dots, but cultures with reduced Sis1 had more small fluorescent dots than cultures with overexpressed Sis1. The differences in predominant fluorescent aggregate types were obvious enough by eye that three investigators were able to distinguish cultures with and without lowered Sis1 blind in more than 6 independent subclones of pTDP-43-YFP transformants. (B) TDP-43-GFP remains largely in the pellet at low and high Sis1 levels. Transformants selected on SD-Ura without doxycycline were inoculated into SRaf/Gal-Ura either without doxycyline (DOX -) to induce TDP-43-GFP with a high level of Sis1, or with 8.5 μg/ml doxycycline, to induce of TDP-43-GFP while depleting Sis1 (DOX +). Cells were harvested at 24 h and lysed. Total (T), supernatant (S) and pellet (P) fractions from lysates were separated by centrifugation at 90,000 rpm for 30 min, resolved with PAGE and immunoblotted with anti-TDP-43 antibodies. Membranes were stripped and reprobed with anti-Sis1 antibodies, and as a control, anti-Pgk1 antibodies for soluble proteins.

Not only does overexpression of Sis1 inhibit toxicity of polyQ and Pin4C but it also dramatically reduces aggregation of these proteins [44,49]. In contrast, TDP-43 formed large aggregates even in the presence of overexpressed Sis1. However, the frequency of small aggregates was influenced by the level of Sis1: when the level of Sis1 was reduced, TDP-43-YFP formed more smaller aggregates that appeared in addition to larger aggregates that were comparable in size and number to that seen with the higher level of Sis1 (Fig 5A). In this experiment, Sis1 expression was controlled via a *TET* promoter, whereby the level of Sis1 was above normal in the absence of doxycycline and below normal in the presence of doxycycline. In control cultures with endogenous Sis1 but no *TET* controlled Sis1 (GF820), aggregation patterns of overexpressed TDP-43-YFP were not affected by doxycycline.

Despite the above aggregation pattern differences, no reproducible differences were seen in the level of soluble vs. insoluble TDP-43 in the presence or absence of Sis1 overexpression. About 55–60% of TDP-43 was found in the pellet whether or not Sis1 was overexpressed (Fig 5B).

### There is no direct evidence for a physical association between Sis1 and TDP-43 aggregates

**Fig 6:**
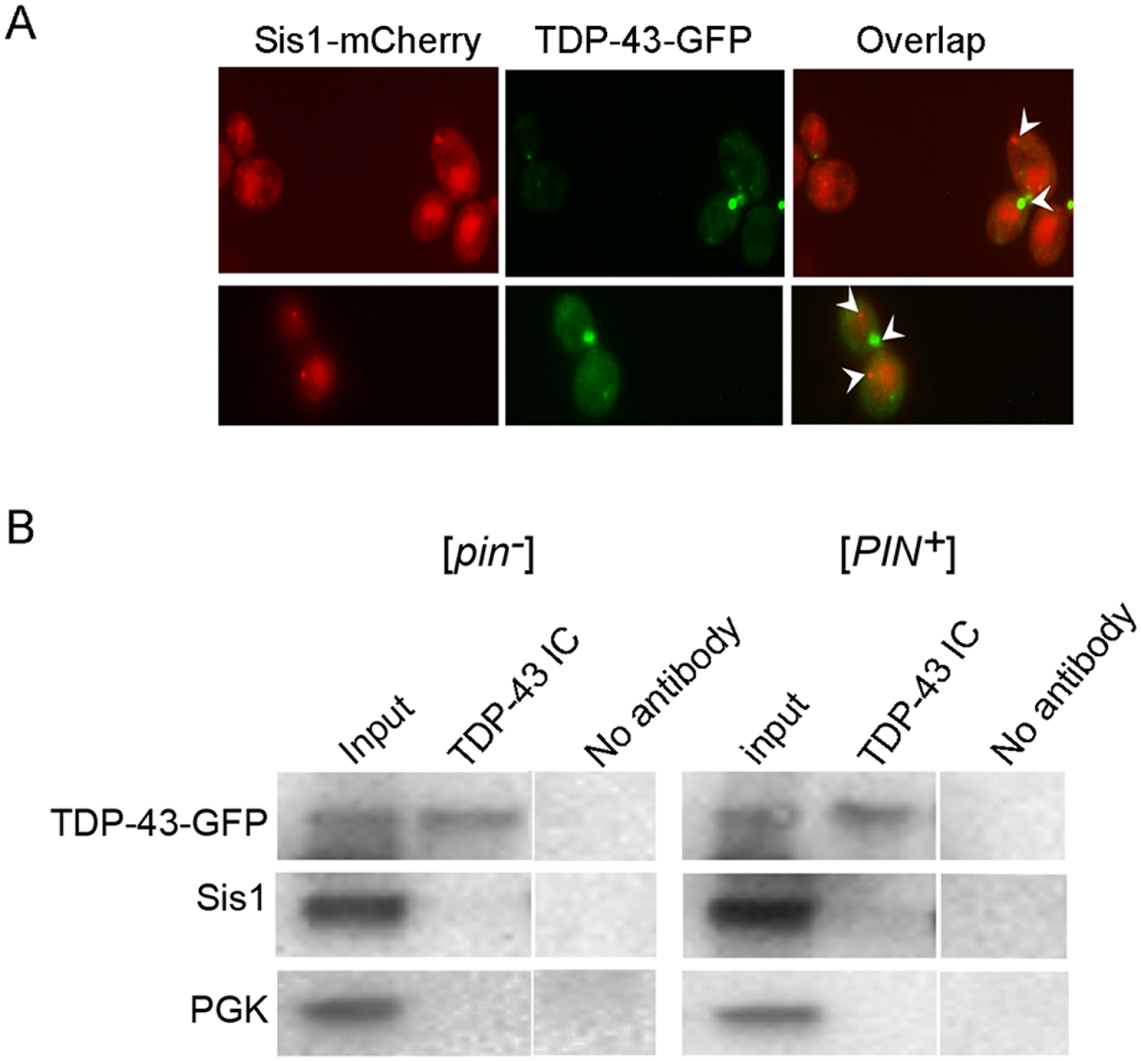
Sis1 and TDP-43 are not physically associated. (A) Cytoplasmic aggregates formed by Sis1-mCherry and TDP-43-GFP appear to be independent. [*PIN*^*+*^] cells with endogenous Sis1 tagged with mCherry (L3478) were transformed with p*GAL1-TDP-43-GFP* (p2288). Galactose to 2% was added to cells grown overnight in 2% raffinose and cells were imaged after 4 h in the mCherry and GFP channels. GFP and mCherry dots (arrow heads) failed to show co-localization. (B) Immunocapture of overexpressed TDP-43-GFP did not co-capture endogenous Sis1. The interaction between TDP-43-GFP (p2288) and endogenous Sis1 was assayed in [*pin*^-^] (L2910) and [*PIN*^+^] (L1749) cells. Lysates (Input) made after 24 h growth in SRaf/Gal-Ura medium to induce TDP-43-GFP were incubated with (TDP-43) or without (control) anti-TDP-43 antibodies. Material bound to the antibody was collected with magnetic beads with immobilized G-protein. Following wash, bound proteins were eluted and analyzed by electrophoresis and immunoblotting. The same membrane was immunoblotted with anti-TDP-43 antibodies, then reprobed with anti-Sis1 and anti-Pgk1 antibodies.

The finding that the essential chaperone, Sis1, is sequestered by PolyQ and Pin4C aggregates suggested that the lack of functional Sis1 could contribute to PolyQ and Pin4C-mediated toxicity [45,49]. We thus asked if the aggregates formed by overexpression of TDP-43-GFP co-localized with endogenous Sis1 tagged with mCherry (Sis1-mCh). Sis1-mCh is largely diffuse in the nucleus but also forms some cytoplasmic dots [71,72]. However, TDP-43-GFP and Sis1-mCh dots failed to show any co-localization (Fig 6A). We also investigated the interaction of Sis1 and TDP-43 with co-immunocapture. Sis1 failed to associate with TDP-43-GFP captured with TDP-43 antibody (Fig 6B).

### DNAJB1 overexpression, but not reduction, modifies TDP-43 toxicity in primary neurons

**Fig 7:**
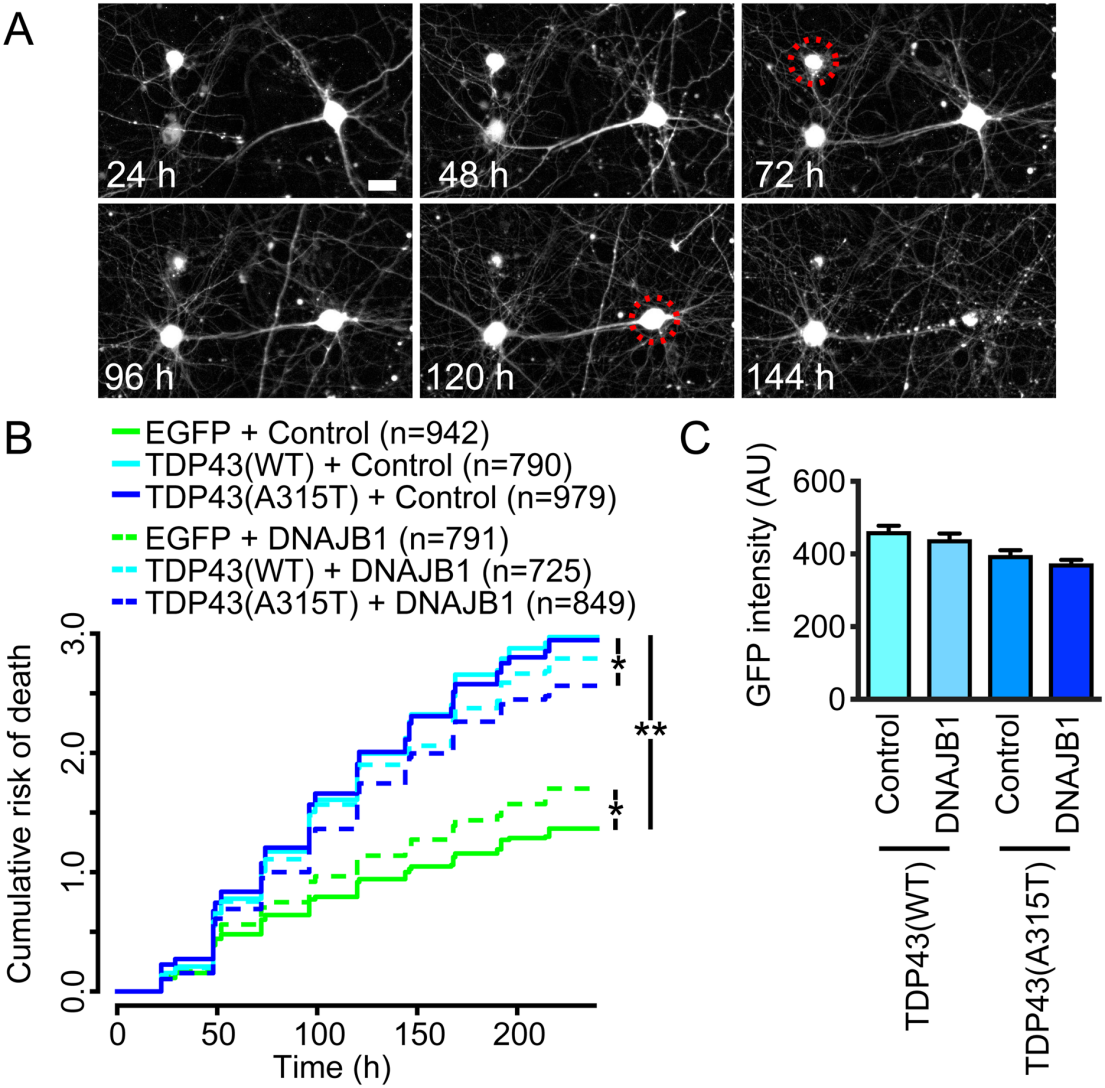
Mammalian DNAJB1 relieves TDP-43 toxicity in primary cortical neurons. Primary rodent cortical neurons were transfected with mApple, and TDP-43(A315T)-EGFP, TDP-43(WT) or EGFP. They were also co-transfected with DNAJB1 or empty vector. (A) Images used to calculate time of death. Cells were imaged at repeated intervals using longitudinal fluorescence microscopy. Time of death (red dotted circles) was estimated conservatively as the last time a neuron was seen alive. Scale bar, 25 μm. (B) Cumulative risk of death plot. While DNAJB1 displays some toxicity in control neurons when overexpressed, it also reduces the risk of death in neurons expressing TDP-43(WT)-EGFP or TDP-43(A315T)-EGFP. *p < 0.005; ** p < 2x10^-16^, by Cox proportional hazards analysis. Results were pooled from three independent experiments. (C) Overexpression of DNAJB1 does not significantly alter the expression of TDP-43. Levels of TDP-43-EGFP and TDP-43(A315T)-GFP were determined in the presence of the plasmid expressing DNAJB1 and the empty vector control, by measuring GFP intensity. Using one-way ANOVA with Tukey’s test to compare all groups, DNAJB1 expression does not have a significant effect on TDP-43-EGFP levels.

There are many Hsp40 chaperones with distinct structures and functions in mammalian cells [73]. One of these proteins, DNAJB1, has the most similarity to the yeast Sis1. To determine if DNAJB1 extended neuronal survival in mammalian neurons, we overexpressed wild-type (WT) and mutant TDP-43 (A315T) linked with familial ALS in rodent primary cortical neurons [8,57]. This model recapitulates several features of human disease, including neurodegeneration, the formation of ubiquitin-positive neuronal aggregates, and cytoplasmic TDP-43 mislocalization in affected cells [3,57]. For these assays, individual neurons expressing WT or mutant TDP-43-EGFP or EGFP alone were monitored at regular intervals for 10 days by fluorescence microscopy, and the time of death for each cell used to create survival or cumulative hazard plots (Fig 7A and 7B). Both WT and mutant TDP-43-EGFP were highly toxic when overexpressed, effectively doubling the risk of death over that of control neurons transfected with EGFP (hazard ratio (HR) 2.02 and 2.06, respectively, p < 2x10^-16^, Cox hazards analysis). Although DNAJB1 expression caused mild toxicity in control neurons (HR 1.24, p = 0.0001), it also extended the survival of neurons transfected with TDP-43(A315T) by 15% (HR 0.85 compared to TDP-43(A315T)-EGFP alone, p = 0.001). In contrast, DNAJB1 co-expression had no significant effect upon TDP-43(WT)-EGFP expressing cells (HR 0.98 compared to TDP-43(WT)-EGFP alone, p = 0.7). Since TDP-43 levels are directly proportional to survival [74], we questioned whether DNAJB1 expression may affect neuronal survival by acting on TDP-43(A315T) levels. However, we detected no significant alterations in TDP-43(WT)-EGFP or TDP-43(A315T)-EGFP levels associated with DNAJB1 expression (Fig 7C). Thus, as in yeast, expression of DNAJB1 partially mitigates TDP-43-dependent toxicity.

To determine if deficiencies in DNAJB1 may potentiate neurodegeneration due to the accumulation of TDP-43, we used siRNA to knockdown DNAJB1 in rodent primary cortical neurons overexpressing TDP-43(WT)-mApple or mApple alone (S6 Fig). Similar to its effects when overexpressed, DNAJB1 knockdown increased the risk of death by 20% in control neurons expressing mApple alone (HR 1.2 compared to scrambled siRNA, p 0.004). Knockdown of DNAJB1 likewise enhanced the risk of death by 21% in neurons overexpressing TDP-43(WT)-mApple (HR 1.21 compared to scrambled siRNA, p = 2.3x10^-5^). These results show that DNAJB1 is essential for maintaining neuronal health, and despite the fact that DNAJB1 can help prevent TDP-43 mediated neurotoxicity, its deficiency does not exacerbate neurodegeneration due to TDP-43 overexpression.

## Discussion

Aggregation of TDP-43 is frequently associated with ALS and other neurological diseases [75–79] and overexpressed human TDP-43 is toxic and forms cytoplasmic aggregates in yeast and mammalian neurons [53,59,64,65]. Our finding in yeast that TDP-43 aggregates inhibit clearance of a misfolded reporter protein, CG* by the ubiquitin proteasome system (UPS) [80], is consistent with the hypothesis that some of the TDP-43 associated toxicity is caused by proteolytic dysfunction. Indeed, failure of proteolysis and the consequent accumulation of misfolded proteins is associated with a variety of diseases [23,24,44]. Furthermore, we found that the effect of TDP-43 on toxicity is correlated with its effect on UPS: TDP-43 is more toxic in [*PIN*^+^] than [*pin*^-^] 74D-694 cells and likewise has a greater effect on UPS in [*PIN*^+^] vs. [*pin*^-^] cells. By analogy, the presence *vs*. absence of heterologous aggregates in mammalian cells may also influence the proteostasis of disease-causing aggregates.

Why and how [*PIN*^+^] effects toxicity and UPS inhibition is unknown. While [*PIN*^*+*^] dramatically enhances aggregation and toxicity of polyQ and Pin4C [44,49], the visible appearance of TDP43 aggregates formed in [*PIN*^*+*^] vs. [*pin-*] cells are indistinguishable. Nonetheless, the fact that [*PIN*^*+*^] prion aggregates sequester some essential chaperones including Sis1 [66] could make [*PIN*^*+*^] cells more vulnerable to TDP-43 overexpression, e.g. the lower level of Sis1 available for the UPS in [*PIN*^*+*^] cells could make cells more sensitive to the inhibitory effect of TDP-43 on the UPS. Indeed, we found that overexpression of Sis1 reduces the deleterious effects of TDP-43 on cell growth, cell shape and UPS inhibition. Several genetic screens in yeast have identified modifiers of TDP-43 toxicity; however, Sis1 was not among them [59,62,81]. The importance of such screens was clearly established when one such modifier, Pbp1, led to the discovery of a new human ALS risk factor, *ATXN2* harboring intermediate length polyglutamine expansions, the human homolog of yeast Pbp1 [59]. Sis1 may have been missed in the overexpression screens because of their high throughput nature. Although the Sis1 effect was weak in the strains used, it was at least as strong as that of several other modifiers that were detected (Hsp104, Pbp1 and hUpf1) (S1 Fig). Since the presence or absence of [*PIN*^*+*^] and other unknown differences between strains alter the toxicity of TDP-43, repeating screens for toxicity modifiers in additional yeast strain backgrounds may reveal new modifiers.

The mechanism by which overexpression of Sis1 rescues TDP-43 toxicity is unknown. Sis1 has a C-terminal substrate binding domain, an N-terminal J domain that regulates the ATPase activity of Hsp70, and a dimerization domain at the end of its C-terminus. It is largely in the nucleus, but is involved with other proteins in nucleocytoplasmic shuttling that transports ubiquitinated proteins to the nucleus for degradation by the proteasome [44]. When cells are subjected to stress, cytoplasmic diffuse Sis1 accumulates in juxtanuclear and peripheral punctate compartments [71], as well as in stress granules, which it helps target for autophagy [72].

Overexpression of Sis1 also rescues yeast from toxicity and inhibition of UPS associated with amyloid aggregates formed upon overexpression of polyQ or Pin4C [44,49]. However, unlike for TDP-43, in these cases Sis1 clearly co-localizes with the aggregates, suggesting that inhibition of Sis1 activity is the source of the toxicity and/or that Sis1 renders the aggregates non-toxic. Indeed, in these cases overexpression of Sis1 inhibits aggregate formation. In contrast, TDP-43 formed large aggregates independently of the Sis1 level, although there were also numerous small TDP-43 aggregates at reduced levels of Sis1. Possibly Sis1 helps to shear small TDP-43 aggregates so that with lower levels of Sis1 the smaller aggregates continue to grow without being sheared and become visible.

In addition, Sis1 overexpression rescues cells from toxicity associated with overexpression of Rnq1 in [*PIN*^+^] cells [50]. In that case, Sis1 overexpression caused a reduction in small soluble RnqQ1 oligomers that were presumed to be the toxic species. Also, Sis1 has been shown to help shear prion aggregates reducing the size of their detergent resistant oligomers [37,38,66,71]. No such effects of Sis1 on TDP-43 oligomer size were detected in our study.

Thus, the mechanism by which Sis1 rescues cells from TDP-43 toxicity appears to be distinct from its rescue of polyQ, Pin4C or Rnq1 toxicity. Previous studies implicated a direct effect of a different HSP40 chaperone, DNAJB6 (a member of the DNAJB7 and 8 family) on TDP-43 via interactions with the TDP-43 C-terminal domain [82,83]. In contrast, we did not detect any direct interaction between TDP-43 and Sis1 (homolog of DNAJB1 and part of the B2, B4, B5, B9 and B11 HSP40 family). Possibly TDP-43 causes toxicity and inhibits UPS for reasons unrelated to Sis1. Overexpression of Sis1 could still rescue by enhancing UPS through an independent mechanism. In support of this hypothesis, Sis1 overexpression in the presence of TDP-43 enhanced UPS activity above that seen in cells without TDP-43 (Fig 4).

Our findings that overexpression of the human homolog of Sis1, DNAJB1, reduced TDP-43 A315T toxicity in primary cortical neurons suggests that the mechanism by which Sis1 reduces cell death is conserved. Indeed, other Hsp40 chaperones have also been reported to affect the cellular formation of amyloid protein aggregates in mammalian cells [83–87]. The magnitude of the Sis1 effect seen here is similar to that seen with other genetic modifications (i.e. *DBR1* knockdown) [88].

Mutations in different Hsp40 family member, DNAJB6, have been shown to cause limb-girdle muscular dystrophy (LGMD) and to inhibit induced nuclear and cytoplasmic TDP-43 aggregation induced by stress, although no effect on toxicity was found [82,83,89]. Our new results show that Sis1 (and in neurons its mammalian homolog, DNAJB1) reduces toxicity arising from TDP-43 accumulation, and furthermore, this occurs in the absence of a direct stressor such as heat. It remains to be determined if the protection afforded by Sis1, and DNAJB1 in mammalian cells, can be an effective means of extending neuronal survival in ALS and in related conditions characterized by cytoplasmic mislocalization and aggregation of DNA/RNA binding proteins such as TDP-43.

## Methods

### Strains and plasmids

Yeast strains and plasmids used in this study are listed in Tables 1 and 2, respectively. In L3478 the C-terminus of *SIS1* was endogenously tagged with *mCherry* by screening L1749 transformants of a PCR *mcherry-HIS5* amplified fragment from p2268 from SD-His plates for the Sis1-mCherry signal. In L3491, the nuclear marker *HTB1* was *mCherry* tagged by transforming L1749 with Cla1 linearized p2278 (pRS305-*ADH1-HTB1-mCherry*). Isogenic [*pin*^-^] versions of strains were obtained on YPD plates with 5 mM GuHCl and were confirmed to be [*pin*^*-*^] by checking for lack of aggregates after transforming with an Rnq1-GFP plasmid [90].

**Table 1:** Yeast strains used in this study.

Strains	Description	Reference
74D-694	*MAT***a** *ade1-14 ura3-52 leu2-3*,*112 trp1-289 his3-200*	[91]
L2910	74D-694 [*psi*^*-*^] [*pin*^*-*^]	[91]
L1749	74D-694 [*psi*^*-*^] [*PIN*^*+*^]	[91]
BY4741	*MAT****a*** *his3Δ1 leu2Δ0 met15Δ0 ura3Δ0*	
L3270	BY4741 [*psi*^*-*^] [*PIN*^*+*^]	[91]
L3269	BY4741 [*psi*^*-*^] [*pin*^*-*^]	[91]
L2389	74D-694 [*psi*^*-*^] [*pin*^*-*^], *hsp104*::*LEU2*	[91]
L3478	74D-694 [*psi*^*-*^] [*PIN*^*+*^] *SIS1-mCherry*::*HIS5*	This study
L3497	[*pin*^-^] version of L3478	This study
L3491	74D-694 [*psi*^*-*^] [*PIN*^*+*^] *HTB1-mCherry*::*LEU2*	This study
L3496	[*pin*^*-*^] version of L3491	This study
GF820	W303 [*PIN*^*+*^] *MAT***a** *leu2-3*,*112 trp1-1 can1-100 ura3-1 ade1-14 his3-11*,*15*	From S. Lindquist, MIT
Y2182	W303 [*PIN*^+^] *sis1Δ*::*LEU2* p*TET*^*R*^*-SIS1*	[37]
L3375	[*pin*^*-*^] version of GF820	This study
1001	*MAT****α*** *kar1-1 SUQ5 ade2-1 his3-202 leu2-1 trp1-63 ura3-52* [*PSI*^*+*^]	[92]
1014	*MAT****α*** *kar1-1 SUQ5 ade2-1 his3-202 leu2-1 trp1-63 ura3-52 SSA1-21 ssa2Δ* [*psi*^*-*^]	[92]
L3504	[*psi*^*-*^] version of 1001	This study
64D-694	*MAT****a*** *adel-l4 trpl-289 leu2-3*,*112 ura3-52 lys9-A21*	[34]
L2642	*64D-694 MAT****α*** *ade1-14 ura3-52 leu2-3*,*112 trp1-289 lys9-A21* [*psi*^*-*^] [*pin*^*-*^]	[34]

**Table 2:** Plasmids used in this study.

Plasmids	Description	Reference
p484	pYES2-*GAL (URA3)*	[50]
p1331	pCM184-*TET*^*R*^ *(HIS3)*	[93]
p1369	pRS316-*GAL1-SSA1 (URA3)*	From S. Lindquist, MIT
p1752	pRS416-*GAL1-YFP* (*URA3*)	[50]
p1759	pYES2-*GAL-SIS1* (*URA3*)	[50]
p1764	pAG416 *GAL1-ccdB-GFP (URA3)*	Addgene plasmid 14195
p1767	pYES3-*GAL-SIS1* (*TRP1*)	[50]
p1285	pRS316 *GAL1-HSP104 (URA3)*	From S. Lindquist, MIT
pBYO11	Yeast Gateway expression vector with ARS1, CEN4, Gal1-10 promoter *(URA3)*	From S. Lindquist, MIT
p2228	pBYO11 *GAL1-PBP1 (URA3*)	From A. Gitler, Stanford Univ.
p1369	pBYO11 *GAL1-SSA1 (URA3*)	From S. Lindquist, MIT
p2292	pYES2ct / hUPF1 (URA3)	[53,54]
p1768	pYES3-*GAL (TRP1)*	[50]
p2039	pAG426 *GAL1-ccdB (URA3)*	Addgene plasmid 14155
p2041	pRS426 GAL1-TDP43-GFP	Addgene plasmid 27467
p2042	pRS416-*GAL1-TDP-43-YFP* (*URA3*)	Addgene plasmid 27447 [60]
p2055	pAG426-*GAL1-TDP-43 (URA3)*	This study
p2368	pAG415-*GAL1-TDP-43 (LEU2)*	This study
p2275	pAG414-*GAL1-TDP-43* (*TRP1*)	This study
p2173	pAG415-*GAL1-TDP-43-DsRed (LEU2)*	This study
p2245	pAG415 *GAL1-ccdB* (*LEU2*)	Addgene plasmid 14145
p2154	pRS413-GAL-ΔssCPY*-GFP (pGAL-CG*) (*HIS3*)	[44]
p2273	pDONR221-*TDP-43* with stop codon	This study
p2367	pDONR221-TDP-43 with no stop codon	This study
p2187	pDONR221-TDP-43-YFP	Addgene plasmid 27470
p2186	pAG413-*GAL1-ccdB* (*HIS3*)	Addgene plasmid 14141
p2189	pAG414-*GAL1-ccdB* (*TRP1*)	Addgene plasmid 14143
p2302	pAG415-*GAL1-ccdB-DsRed* (*LEU2*)	Addgene plasmid 14361
p2195	pAG413-*GAL1-TDP-43-YFP* (*HIS3)*	This study
p1185	*CUP1-RNQ1-GFP (TRP1)*	[90]
p1576	pCM184 Gateway expression vector with *TET*^*R*^	This study
p2223	p*TET*^*R*^ *-TDP-43-YFP*	This study
p2201	*eIF4A*	[61]Harvard Plasmid Repository (HsCD00412730
p2203	pCNA-*DNAJB1-V5*	[94]
p2218	pCM184-*TET*^*R*^*-SIS1 (TRP1)*	[37]
p2268	*pGW1-mApple*pFA6a-link-mCherry-HIS5	Addgene plasmid 44643 [95]
p2278	pRS305-*ADH1*-*HTB1-mCherry* (*LEU2)*	[61]
p2288	pRS416 *GAL1-TDP-43-GFP (URA3)*	This study
	*GW1-DNAJB1pGW1-mApple*	[74]
	*pGW1-TDP43(A315T)-EGFP pGW1-DNAJB1*	[74]
	*pGW1 pGW1-TDP43(A315T)-EGFP*	[74]
	*pGW1-EGFP pGW1*	[74]
	*pGW1-EGFP*	[74]

Unless otherwise stated, all overexpression plasmids were driven by *GAL1*. The TDP-43 entry clones (p2273 and p2367) were made by BP reactions between pDONR221 and a PCR amplified TDP-43 fragment with (p2273) or without a stop codon (p2367) using Gateway Technology [96] and the *TDP-43* fragment in p2273 was further transferred to p2039, p2189 and p2245 to build p2055 (pAG426 *GAL1-TDP43*), p2275 (pAG414 *GAL1-TDP43*) and p2368 (pAG415-*GAL1-TDP43)*, respectively. pDONR221-TDP-43 without stop codon (p2367) was used as a donor plasmid to build p*GAL1-TDP-43-DsRed* (p2173) on a destination vector (p2302). Plasmid p2195 (pAG413-*GAL1-TDP43-EYFP*) was constructed by a LR reaction between pDONR221-TDP-43-EYFP (p2187) and p2186 (pAG413-*GAL1-ccdB*). Plasmid p2288 (pRS416 *GAL1-TDP-43-GFP*) was generated by switching *TDP-43-YFP* in p2042 (pRS416 *GAL1-TDP43-YFP*) with *TDP-43-GFP* from p2041 (pRS426 *GAL1-TDP43-GFP*). *TET* promoter driven TDP-43-YFP (p2223) was constructed on pCM184, which was transformed into a Gateway expression vector (p1576).

### Cultivation procedures

Yeast strains were cultivated using standard media and growth conditions [97]. Rich media contained 2% dextrose (YPD). Synthetic complete media contained all amino acids except for those used for selection and 2% dextrose (SD); 2% glycerol (SG); 2% galactose (SGal); 2% raffinose (SRaf) or 2% galactose + 2% raffinose (SGal/Raf). To avoid collecting suppressor mutants that reversed TDP-43 toxicity, p*GAL1*-*TDP-43* transformants were routinely plated and patched on plasmid selective SD medium where TDP-43 was not expressed. Patches were then replica-plated onto SG. Cells that failed to grow on SG were dropped from further study because they were petites. For analysis of growth, non-petite transformants taken from SD plates and suspended in water were normalized to an OD600 of 1.5. Then, 15 μl of 10X serially diluted cell suspensions were spotted on plasmid selective SD and SGal.

### Measuring cell death

Cells containing the p*GAL1-TDP-43* or control plasmids grown overnight in selective SRaf were resuspended and grown in SGal/Raf for 72 h to allow for TDP-43 overexpression. Then cells were resuspended in 1/10^th^ volume TE and 1.5 μl were mixed on slides with 1.5 μl of 0.4% trypan blue. Different fields of the cells were then photograph under a Nikon Eclipse fluorescent microscope. The number of blue vs. unstained cells were counted blind. Between 820 and 1106 cells were counted for each sample.

### Animal work

All vertebrate animal work was approved by the Committee on the Use and Care of Animals at the University of Michigan (protocol # PRO00007096). All experiments were carefully planned so that we use as few animals as possible. Pregnant female wild-type, non-transgenic Long Evans rats (Rattus norvegicus) were housed singly in chambers equipped with environmental enrichment. They were fed ad libitum a full diet (30% protein, 13% fat, 57% carbohydrate; full information available at www.labdiet.com), and cared for by the Unit for Laboratory Animal Medicine (ULAM) at the University of Michigan. Veterinary specialists and technicians in ULAM are trained and approved in the care and long-term maintenance of rodent colonies, in accordance with the NIH-supported Guide for the Care and Use of Laboratory Animals. All rats were kept in routine housing for as little time as possible prior to euthanasia and dissection, minimizing any pain and/or discomfort. Pregnant dams were euthanized by CO2 inhalation at gestation day 20. For each animal, euthanasia was confirmed by bilateral pneumothorax. Euthanasia was fully consistent with the recommendations of the Guidelines on Euthanasia of the American Veterinary Medical Association and the University of Michigan Methods of Euthanasia by Species Guidelines. Following euthanasia, the fetuses were removed in a sterile manner from the uterus and decapitated. Primary cells from these fetuses were dissected and cultured immediately afterwards.

### Primary cortical neuron methods

Mixed primary cortical neurons were isolated from E20 rat pups as described previously [98] and transiently transfected on day 4 *in vitro* (DIV4) with plasmids encoding TDP-43 variants, DNAJB1, or siRNA targeting DNAJB1 (Dharmacon ON-TARGETplus Rat Dnajb1 (361384) siRNA—SMARTpool) using Lipofectamine 2000 (Invitrogen) [74]. We then tracked neuronal survival using a system of fully automated fluorescence microscopy [8,74,99]. Background was digitally subtracted from raw images and the adjusted images were stitched together in Fiji. Stitched images were sequenced and registered (aligned) using the multistackreg plugin in Fiji, and neuron survival determined by custom-designed code written in Python. In this method cell death is marked by disruption of the cell membrane, loss of neuronal processes, or rounding of the cell body. We plotted the cumulative risk of death for neurons in each population, and compared survival between groups using Cox proportional hazards analysis. All statistical analyses were performed in R using the survival analysis package.

Imaging was accomplished using a Nikon TiE-B inverted microscope equipped with PerfectFocus3, a high-numerical aperture 20X objective lens and a 16-bit Andor iXon electron multiplied ultra-cooled charge-coupled device digital camera. A Lambda XL Xenon lamp (Sutter) illuminated the samples via a 5-mm liquid light guide. All x- and y- stage movements were performed using an ASI 2000 stage and rotary encoders. An environmental chamber that maintained 37°C and 5% CO_2_ was used for all experiments. All movements of the stage, shutters, and PerfectFocus3 system were controlled by BeanShell code from μManager, publicly available software that runs in ImageJ.

### Immunocytochemistry

Rodent primary cortical neurons were isolated and transfected on DIV4 as described above. Forty-eight hours later the cells were rinsed twice in PBS, then fixed in 4% paraformaldehyde for 10 min. Following 2 more rinses, neurons were permeabilized with 0.1% Triton X-100 in PBS for 20 min at room temperature, equilibrated with 10 mM glycine in PBS for 10 min at room temperature, then blocked in 0.1% Triton X-100, 3% BSA and 0.2% goat serum in PBS for 1 hour at room temperature. Primary antibodies against DNAJB1 (Abcam ab69402, rabbit polyclonal anti-Hsp40, 1:100) were added directly to the block and the samples incubated overnight at 4°C. All cells were rinsed twice quickly and 3 times for 10 min with PBS, then placed back in block solution containing the appropriate secondary antibodies (Donkey anti-rabbit Cy5, Jackson ImmunoResearch, 711-175-152, whole IgG) at a dilution of 1:250. The cells were rinsed twice quickly in PBS, and 3 times for 10 min each in PBS containing Hoescht dye (33342, Invitrogen) at 100 nM, then twice more in PBS before imaging by automated microscopy.

### Measuring protein levels

Equal amounts of total proteins in precleared lysates were analyzed by Western blot as described previously [100]. To analyze TDP-43 aggregates on semi-denaturing Detergent Agarose Gel electrophoresis (SDD-AGE) proteins were extracted from cells expressing *GAL1-*controlled TDP-43-GFP [101,102]. Around 60 μg of crude lysate was treated with 2% sarkosyl sample buffer (25 mM Tris, 200 mM glycine, 5% glycerol, and 0.025% bromophenol blue) for 7 min at room temperature and electrophoretically resolved in a horizontal 1.5% agarose gel in a standard tris/glycine/SDS buffer, transferred to a PVDF membrane and probed with indicated antibodies. To visualized monomer TDP-43, the crude lysates were boiled at 95°C for 5 min in 2% SDS sample buffer containing 80 mM dithiothreitol (DTT) prior to electrophoresis. Blots were developed with TDP-43 rabbit polyclonal antibody from Proteintech Group (Rosemont, IL); Sis1 antibody kindly provided by E. Craig (Madison, WI); Sup35C monoclonal antibody (made in our lab by Viravan Prapapanich). GFP and PGK antibodies were purchased from Roche Applied Science (Indianapolis, IN) and Novex (Frederick, MD), respectively.

To compare levels of proteins in supernatant vs. pellet, normalized cell lysates [103] (300 μl at a concentration of 1 mg of protein /ml) were centrifuged at 90,000 rpm for 30 min at 4°C to separate supernatant and pellet fractions. After the supernatant was removed and saved, pellets were washed with the lysate buffer containing a protease inhibitor cocktail, recentrifuged at 90,000 rpm for 10 min and resuspended in 300 μl of lysate buffer. Boiled proteins in total (T), supernatant (S) and pellet (P) fractions were resolved by PAGE that was immunoblotted with anti-TDP-43. The blots were then stripped and reprobed with anti-Sis1 and the loading control anti-Pgk1.

### Visualization of aggregates and co-localization studies

Aggregates formed in cells by fluorescently labeled proteins were examined with a Nikon Eclipse E600 fluorescent microscope (100X oil immersion) equipped with FITC, YFP and mCherry (respectively, chroma 49011, 49003 and 49008) filter cubes. Cells were sometimes fixed with formaldehyde 3.7% final concentration for 15 minutes before washing with 0.1M potassium phosphate (pH 7.5) and were imaged over the next few days (http://openwetware.org/wiki/McClean:_Fixation_of_Yeast,Bisaria_Protocol)). Fixed and unfixed cells looked identical in shape and GFP and mCherry fluorescence in control experiments. Cells were always examined and photographed in the mCherry channel before being exposed to FITC excitation and all experiments controlled for very bright GFP fluorescence that also appeared in the mCherry channel even in the absence of an mCherry tag.

### Immunocapture

Cells containing TDP-43-GFP (p2288) made after 24 h growth in SRaf/Gal-Ura medium to induce TDP-43-GFP were harvested, and washed in ice cold water. As described previously [100], lysates were made by vortexing 800 μl cells resuspended in LB2 buffer [40 mM Tris-HCl (pH 7.5), 150 mM KCl, 5 mM MgCl2, 10% glycerol] containing anti-protease cocktail and PMSF. Then 500 μl of the precleared lysate containing 0.5–1.0 mg/ml proteins were incubated with 2 μl of TDP-43 antibody for 2 h on ice; samples were then mixed with 50 μl magnetic beads with immobilized G protein (Miltenyi Biotec Inc., San Diego, CA) and incubated on ice for 1 h. Nonspecifically bound proteins were removed in the following order of washing steps. Beads were washed with 1.0 ml of each of the following solutions at room temperature: LB2 with 1% Triton X-100 with LB2, 210 mM KCl, 1% Triton X-100; LB2 with 1% Triton X- 100; LB2; LB1 [40 mM Tris-HCl (pH 7.5), 50 mM KCl, 5 mM MgCl2, 5% glycerol]; 20 mM Tris-HCl (pH 7.6). Proteins were eluted with hot sample buffer (50 mM Tris-HCl, pH 6.8, 5% glycerol, and 0.05 and 2% β-mercaptoethanol), and then they were analyzed by electrophoresis and immunoblotting. Total (Input) protein was boiled at 95°C for 5 min in 2% SDS sample buffer containing 80 mM DTT.

## Supporting information

S1 Fig Comparison of growth inhibition caused by TDP-43 overexpression in the presence of different toxicity modifiers (overexpression of Sis1, Hsp104, Pbp1, and hUpf1) in [*PIN*^*+*^] and [*pin*^*-*^] versions of different yeast strains. [*PIN*^*+*^] and [*pin*^*-*^] versions of 74D-694, W303 and BY4741 were doubly transformed with *GAL1* controlled TDP-43-DsRed, *GAL1* controlled modifier or control empty vector plasmids listed below. Transformants were selected on SD-Leu-Ura plates. Normalized suspensions of cells taken from SD-Leu-Ura were 10X serially diluted in water and 15 μl were spotted on SD-Leu-Ura (dextrose), and 2% Gal-Leu-Ura (galactose) plates, which were photographed after 3 (dextrose) or 5 (galactose) days of incubation at 30°C. We show hUpf1 because it had a bigger effect than the yeast homologue Ecm32 found in the initial screen [64].
v/v: p2302 (pAG415 *GAL1-ccdB-DsRed*, *LEU2*) / p484 (*GAL1*, *URA3*)
TDP-43/v: p2173 (pAG415 *GAL1-TDP-43-DsRed*, *LEU2*) / p484 (*GAL1*, URA3)
TDP-43/Sis1: p2173 (pAG415 *GAL1-TDP-43-DsRed*, *LEU2*) / p1759 (*GAL1-SIS1*, *URA3*)
v/Sis1: p2302 (pAG415 *GAL1-ccdB-DsRed*, *LEU2*) / p1759 (*GAL1-SIS1*, *URA3*)
v/v: p2302 (pAG415 *GAL1L-ccdB-DsRed*, *LEU2*) / p484 (*GAL1*, *URA3*)
TDP-43/v: p2173 (pAG415 *GAL1-TDP-43-DsRed*, *LEU2*) / p484 (*GAL1*, *URA3*)
TDP-43/Hsp104: p2173 (pAG415 *GAL1-TDP-43-DsRed*, *LEU2*) / p1285 (*GAL1-HSP104*, *URA3*)
v/Hsp104: p2302 (pAG415 *GAL1-ccdB-DsRed*, *LEU2*) / p1285 (*GAL1-HSP104*, *URA3*)
v/v: p2302 (pAG415 *GAL1-ccdB-DsRed*, *LEU2*) / p484 (*GAL1*, *URA3*)
TDP-43/v: p2173 (pAG415 *GAL1-TDP-43-DsRed*, *LEU2*) / p484 (*GAL1*, *URA3*)
TDP-43/hUpf1: p2173 (pAG415 *GAL1-TDP-43-DsRed*, *LEU2*) / p2292 (*GAL1-hUpf1*, *URA3*)
v/hUpf1: p2302 (pAG415 *GAL1-ccdB-DsRed*, *LEU2*) / p2292 (*GA1L-hUpf1*, *URA3*)
v/v: p2245 (pAG415 *GAL1-ccdB*, *LEU2*) / p484 (*GAL1*, *URA3*)
TDP-43/v: p2368 (pAG415 *GAL1-TDP-43*, *LEU2*) / p484 (*GAL1*, *URA3*)
TDP-43/hUpf1: p2368 (pAG415 *GAL1-TDP-43*, *LEU2*) / p2292 (*GAL1-hUpf1*, *URA3*)
TDP-43/Sis1: p2368 (pAG415 *GAL1-TDP-43*, *LEU2*) / p1759 (*GAL1-SIS1*, *URA3*)
v/v: p2302 (pAG415 *GAL1-ccdB-DsRed*, *LEU2*) / p484 (*GAL1*, *URA3*)
TDP-43/v: p2173 (pAG415 *GAL1-TDP-43-DsRed*, *LEU2*) / p484 (*GAL1*, *URA3*)
TDP-43/Pbp1: p2173 (pAG415 *GAL1-TDP-43-DsRed*, *LEU2*) / p2228 (*GAL1-PBP1*, *URA3*)
(PDF)

S2 Fig Overexpression of TDP-43 causes elongated cell shape. [*PIN*^+^] and [*pin*^-^] versions of 74D-694 (A), BY4741 (B) and W303 (C) carrying p*GAL1-TDP-43-DsRed* (p2173) or pAG415 *GAL1-ccdB-DsRed* (p2302) were grown on 2% galactose plates for 4 days and then imaged at the same magnification. Shown are representative fields of many fields photographed and examined.
(PDF)

S3 Fig Cell elongation is associated with TDP-43 protein levels. Transformants of [*pin-*] 74D-694 with p*TET*^*R*^*-TDP-43-YFP* (p2223) were selected on SD-Trp supplemented with doxycycline (10 μg/ml). Transformants were then grown in liquid SGal-Trp media with the indicated amount of doxycycline for 24 h and were examined and photographed at same magnification. The levels of TDP-43-YFP were determined by immunoblotting SDS-PAGE gels of normalized cell lysates probed with anti-TDP-43 antibodies, and anti-Pgk1 antibodies as an internal loading control.
(PDF)

S4 Fig Sis1 overexpression does not cure cells of [*PIN*^+^]. [*PIN*^+^] and [*pin*^-^] 74D-694 doubly transformed with p*GAL1-TDP-43-DsRed* (p2173) and p*GAL-Sis1* (p1759) were grown in plasmid selective synthetic liquid media containing 2% galactose and 2% raffinose for 2 days. Cells were then crossed to [*pin*^-^] 64D-694 *MAT****α*** (L2642) bearing plasmid p1185 (p*CUP1-RNQ1-GFP*). Diploids were selected on SD-Leu-Ura-Trp, expressed on SD-Leu-Ura-Trp supplemented with Cu^++^ (50 μg/ml), and examined for Rnq1-GFP dots or diffuse fluorescence, which was diagnostic for the presence or absence, respectively, of the [*PIN*^+^] prion.
(PDF)

S5 Fig Alteration of Hsp104 and Ssa1 levels does not affect ability of Sis1 overexpression to reduce TDP-43 toxicity. A. Deletion of *HSP104* does not prevent Sis1 overexpression from reducing TDP-43 toxicity. Isogenic [*psi*^-^] [*pin*^-^] versions of 74D-694 with (*hsp104Δ*) and without (WT) a deletion of *HSP104* doubly transformed with p2042 (p*GAL1-TDP-43-YFP*) and p1767 (p*GAL1-SIS1*) or vector control p1752 (p*GAL1-YFP*) and p1768 (p*GAL1*) were selected on SD-Ura-Trp plates. Normalized suspensions of cells taken from SD-Ura-Trp were 10X serially diluted in water and 15 μl were spotted on SD-Ura-Trp (dextrose), and 2% SGal-Ura-Trp (galactose) plates which were photographed after 3 (dextrose) or 7 (galactose) days of incubation at 30°C. B. Reduction of Ssa1 activity does not prevent Sis1 overexpression from reducing TDP-43 toxicity. Strain 1014 bearing a deletion of *SSA2* and expressing a dominant negative allele of *SSA1* (*SSA1-21 ssa2Δ)* and its isogenic parent strain, L3504 (WT) were doubly transformed with p*GAL1-TDP-43-DsRed* (p2173), p*GAL-SIS1* (p1759), or vector controls (p2302 or p484). Normalized suspensions of cells taken from plasmid selective SD-Leu-Ura medium were 10X serially diluted in water and 15 μl were spotted on SD-Leu-Ura (dextrose), and 2% Gal-Leu-Ura (galactose) plates, which were photographed after 3 (dextrose) or 5 (galactose) days of incubation at 30°C.
(PDF)

S6 Fig DNAJB1 deficiency does not exacerbate TDP-43 toxicity. Rodent primary cortical neurons were dissected and transfected with plasmids encoding EGFP and TDP-43(WT)-mApple or mApple. In each case, neurons were also transfected with scrambled siRNA or siRNA targeting DNAJB1. (A) Knockdown was validated by immunocytochemistry using antibodies against DNAJB1. Scale bar, 50 μm. (B) Transfection with siRNA against DNAJB1 resulted in a 60% reduction in anti-DNAJB1 antibody reactivity (N = 101 and 62 neurons from Scr and siDNAJB1, respectively. ** p < 0.0001 by the Mann—Whitney U test. (C) In longitudinal assays of neuronal survival, DNAJB1 knockdown enhanced the risk of death by 20% in control neurons expressing EGFP alone and in neurons overexpressing TDP43. * HR 1.20, p 0.004; ** HR 1.21, p 2.3x10^-5^; ^#^ HR 3.18, p < 2x10^-16^, Cox proportional hazards analysis. Results were pooled from two independent experiments.*
(PDF)
