# Supplementary figures and images for: Overexpression of the essential Sis1 chaperone reduces TDP-43 effects on toxicity and proteolysis
Source: PLoS Genet. 2017 May 22;13(5):e1006805. doi: 10.1371/journal.pgen.1006805 (PMC5460882; doi:10.1371/journal.pgen.1006805)

S1 Fig

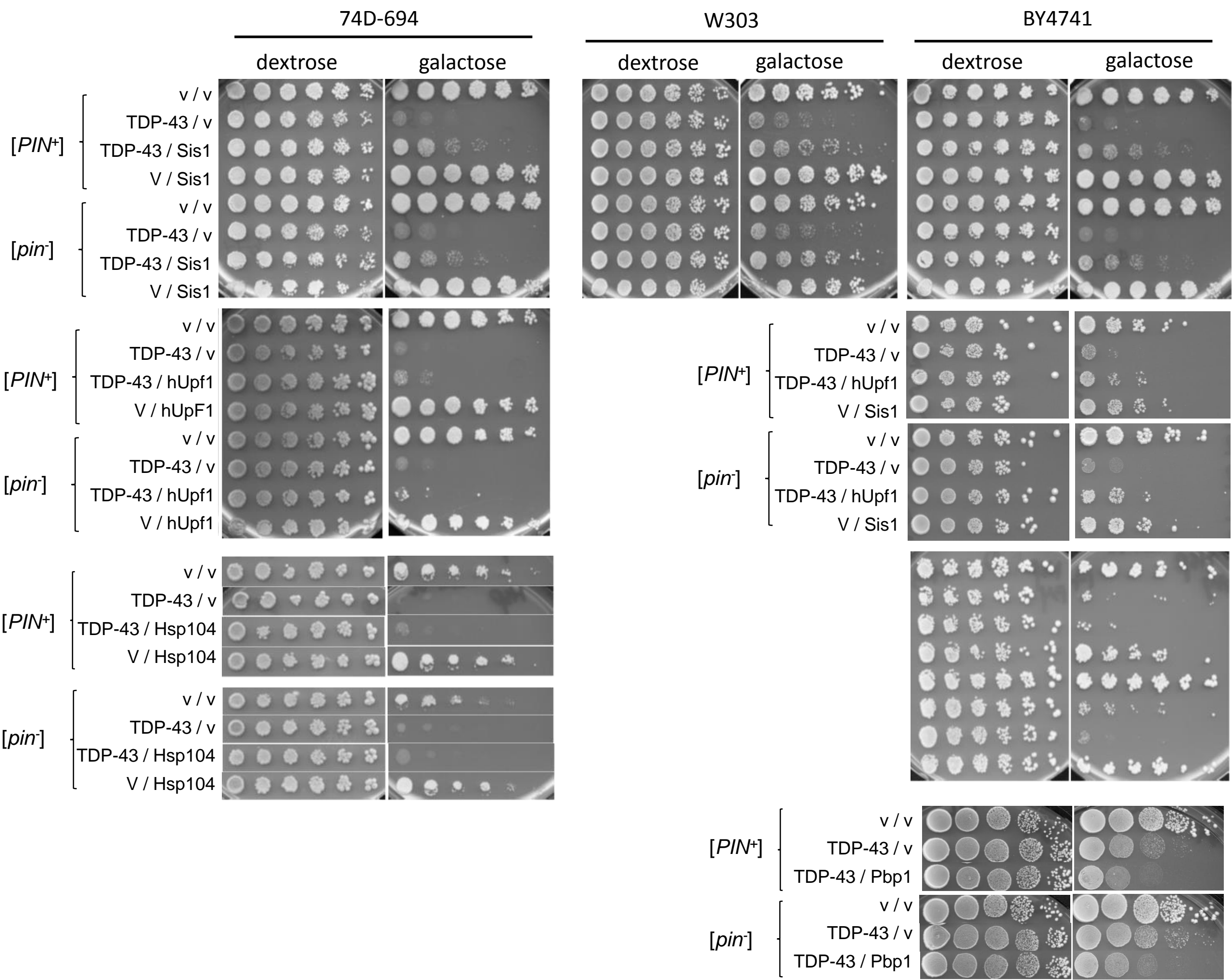

Supplement: S1 Fig — [PIN+] and [pin-] versions of 74D-694, W303 and BY4741 were doubly transformed with GAL1 controlled TDP-43-DsRed, GAL1 controlled modifier or control empty vector plasmids listed below. Transformants were selected on SD-Leu-Ura plates. Normalized suspensions of cells taken from SD-Leu-Ura were 10X serially diluted in water and 15 μl were spotted on SD-Leu-Ura (dextrose), and 2% Gal-Leu-Ura (galactose) plates, which were photographed after 3 (dextrose) or 5 (galactose) days of incubation at 30°C. We show hUpf1 because it had a bigger effect than the yeast homologue Ecm32 found in the initial screen [64]. v/v: p2302 (pAG415 GAL1-ccdB-DsRed, LEU2) / p484 (GAL1, URA3) TDP-43/v: p2173 (pAG415 GAL1-TDP-43-DsRed, LEU2) / p484 (GAL1, URA3) TDP-43/Sis1: p2173 (pAG415 GAL1-TDP-43-DsRed, LEU2) / p1759 (GAL1-SIS1, URA3) v/Sis1: p2302 (pAG415 GAL1-ccdB-DsRed, LEU2) / p1759 (GAL1-SIS1, URA3) v/v: p2302 (pAG415 GAL1L-ccdB-DsRed, LEU2) / p484 (GAL1, URA3) TDP-43/v: p2173 (pAG415 GAL1-TDP-43-DsRed, LEU2) / p484 (GAL1, URA3) TDP-43/Hsp104: p2173 (pAG415 GAL1-TDP-43-DsRed, LEU2) / p1285 (GAL1-HSP104, URA3) v/Hsp104: p2302 (pAG415 GAL1-ccdB-DsRed, LEU2) / p1285 (GAL1-HSP104, URA3) v/v: p2302 (pAG415 GAL1-ccdB-DsRed, LEU2) / p484 (GAL1, URA3) TDP-43/v: p2173 (pAG415 GAL1-TDP-43-DsRed, LEU2) / p484 (GAL1, URA3) TDP-43/hUpf1: p2173 (pAG415 GAL1-TDP-43-DsRed, LEU2) / p2292 (GAL1-hUpf1, URA3) v/hUpf1: p2302 (pAG415 GAL1-ccdB-DsRed, LEU2) / p2292 (GA1L-hUpf1, URA3) v/v: p2245 (pAG415 GAL1-ccdB, LEU2) / p484 (GAL1, URA3) TDP-43/v: p2368 (pAG415 GAL1-TDP-43, LEU2) / p484 (GAL1, URA3) TDP-43/hUpf1: p2368 (pAG415 GAL1-TDP-43, LEU2) / p2292 (GAL1-hUpf1, URA3) TDP-43/Sis1: p2368 (pAG415 GAL1-TDP-43, LEU2) / p1759 (GAL1-SIS1, URA3) v/v: p2302 (pAG415 GAL1-ccdB-DsRed, LEU2) / p484 (GAL1, URA3) TDP-43/v: p2173 (pAG415 GAL1-TDP-43-DsRed, LEU2) / p484 (GAL1, URA3) TDP-43/Pbp1: p2173 (pAG415 GAL1-TDP-43-DsRed, LEU2) / p2228 (GAL1-PBP1, URA3) (PDF) [file pgen.1006805.s001.pdf]

S3 Fig

DOX:                      0.5                      0.05                      0.01 (μg/ml)

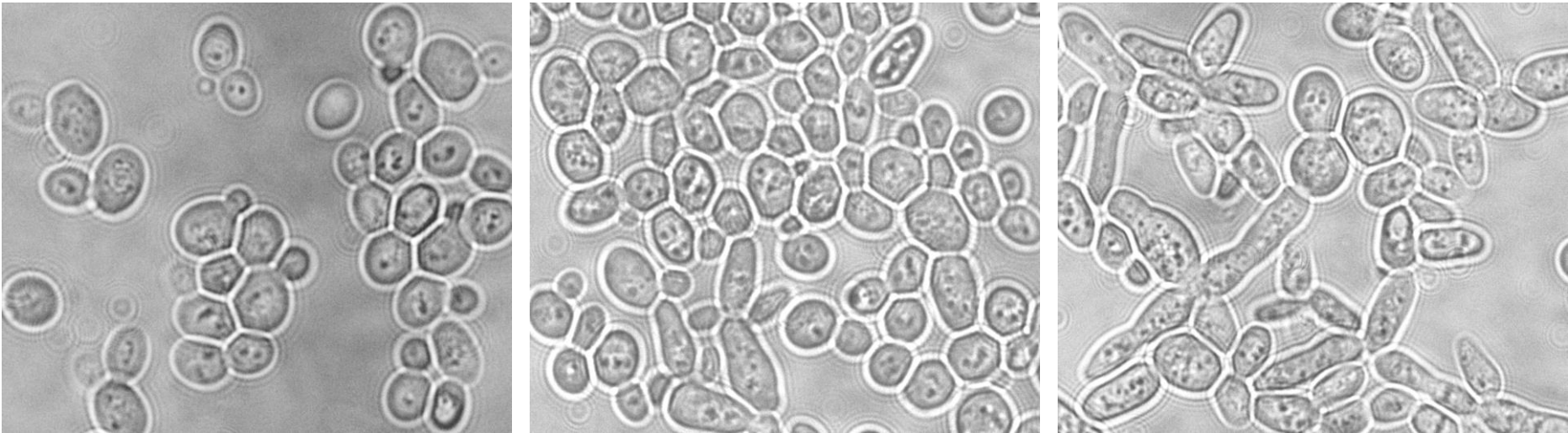

α-TDP-43  
α-PGK

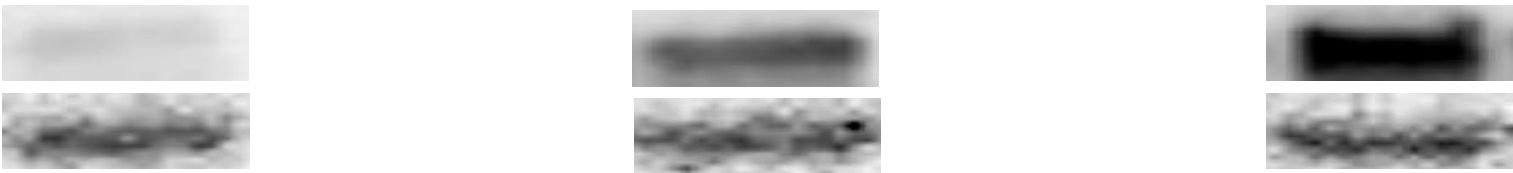

Supplement: S3 Fig — Transformants of [pin-] 74D-694 with pTETR-TDP-43-YFP (p2223) were selected on SD-Trp supplemented with doxycycline (10 μg/ml). Transformants were then grown in liquid SGal-Trp media with the indicated amount of doxycycline for 24 h and were examined and photographed at same magnification. The levels of TDP-43-YFP were determined by immunoblotting SDS-PAGE gels of normalized cell lysates probed with anti-TDP-43 antibodies, and anti-Pgk1 antibodies as an internal loading control. (PDF) [file pgen.1006805.s003.pdf]

S4 Fig

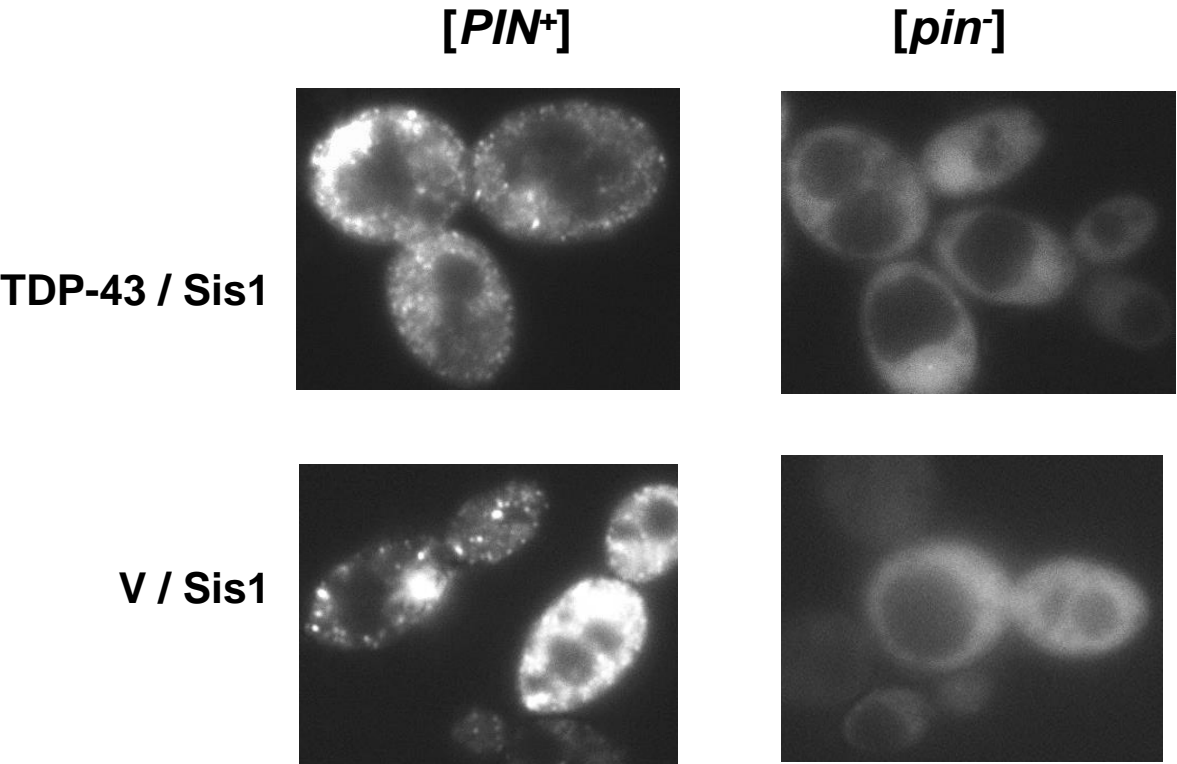

Supplement: S4 Fig — [PIN+] and [pin-] 74D-694 doubly transformed with pGAL1-TDP-43-DsRed (p2173) and pGAL-Sis1 (p1759) were grown in plasmid selective synthetic liquid media containing 2% galactose and 2% raffinose for 2 days. Cells were then crossed to [pin-] 64D-694 MATα (L2642) bearing plasmid p1185 (pCUP1-RNQ1-GFP). Diploids were selected on SD-Leu-Ura-Trp, expressed on SD-Leu-Ura-Trp supplemented with Cu++ (50 μg/ml), and examined for Rnq1-GFP dots or diffuse fluorescence, which was diagnostic for the presence or absence, respectively, of the [PIN+] prion. (PDF) [file pgen.1006805.s004.pdf]

S5 Fig

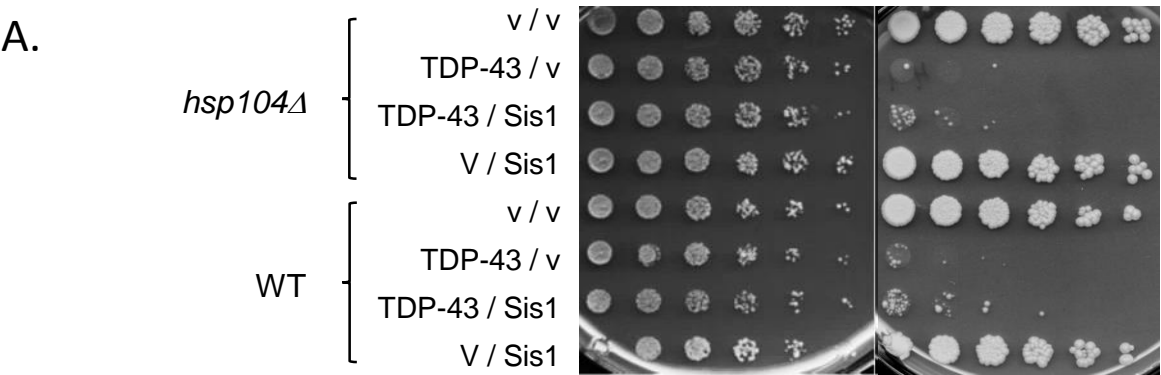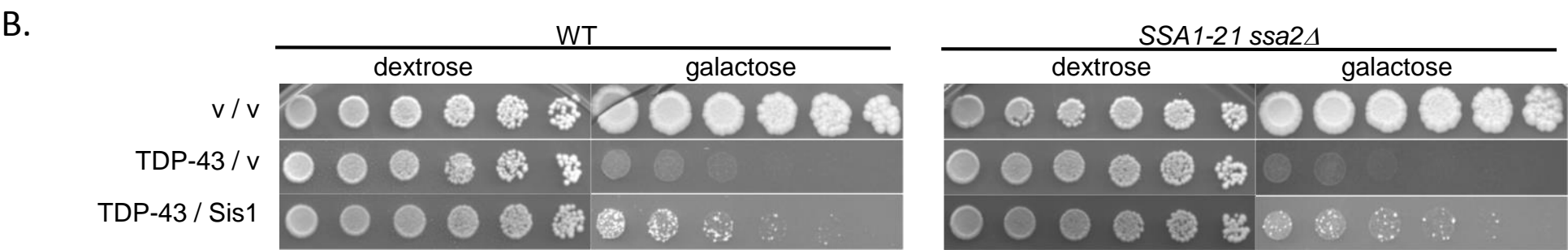

Supplement: S5 Fig — A. Deletion of HSP104 does not prevent Sis1 overexpression from reducing TDP-43 toxicity. Isogenic [psi-] [pin-] versions of 74D-694 with (hsp104Δ) and without (WT) a deletion of HSP104 doubly transformed with p2042 (pGAL1-TDP-43-YFP) and p1767 (pGAL1-SIS1) or vector control p1752 (pGAL1-YFP) and p1768 (pGAL1) were selected on SD-Ura-Trp plates. Normalized suspensions of cells taken from SD-Ura-Trp were 10X serially diluted in water and 15 μl were spotted on SD-Ura-Trp (dextrose), and 2% SGal-Ura-Trp (galactose) plates which were photographed after 3 (dextrose) or 7 (galactose) days of incubation at 30°C. B. Reduction of Ssa1 activity does not prevent Sis1 overexpression from reducing TDP-43 toxicity. Strain 1014 bearing a deletion of SSA2 and expressing a dominant negative allele of SSA1 (SSA1-21 ssa2Δ) and its isogenic parent strain, L3504 (WT) were doubly transformed with pGAL1-TDP-43-DsRed (p2173), pGAL-SIS1 (p1759), or vector controls (p2302 or p484). Normalized suspensions of cells taken from plasmid selective SD-Leu-Ura medium were 10X serially diluted in water and 15 μl were spotted on SD-Leu-Ura (dextrose), and 2% Gal-Leu-Ura (galactose) plates, which were photographed after 3 (dextrose) or 5 (galactose) days of incubation at 30°C. (PDF) [file pgen.1006805.s005.pdf]

S6 Fig

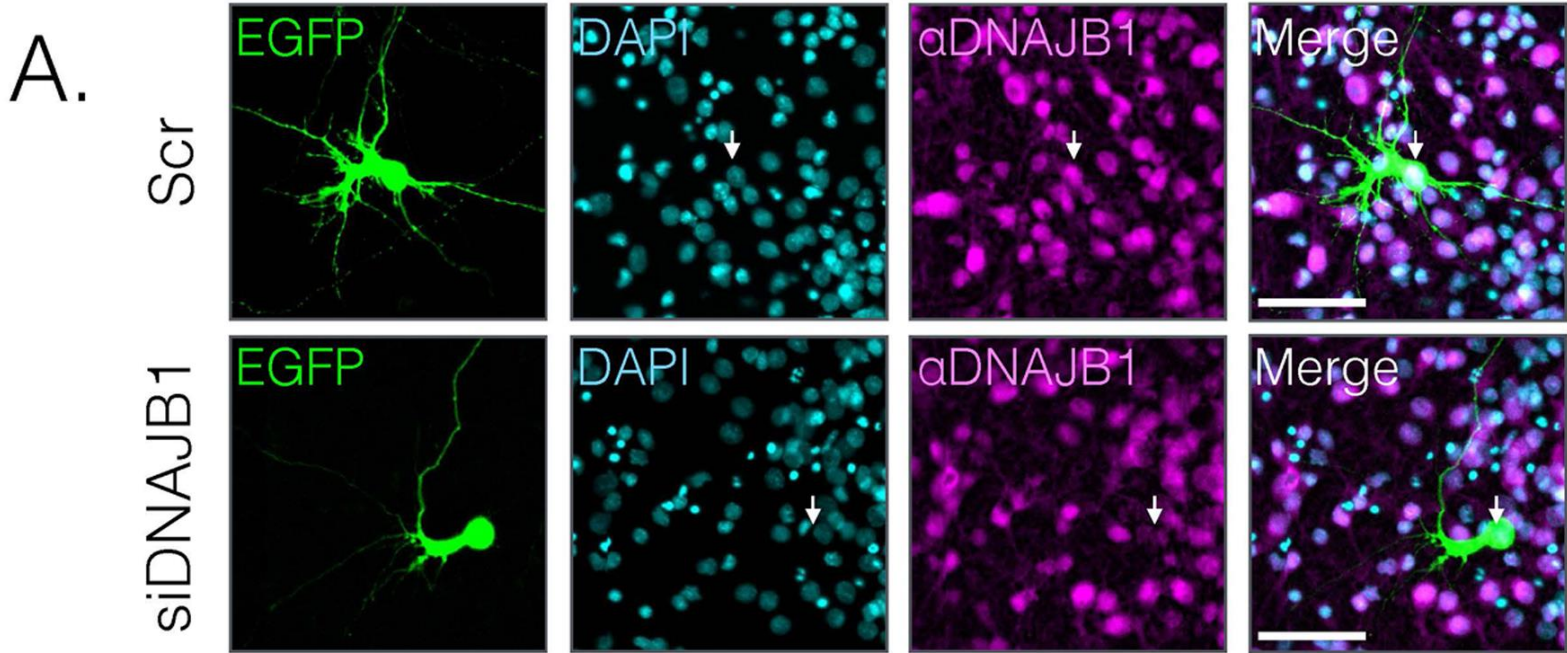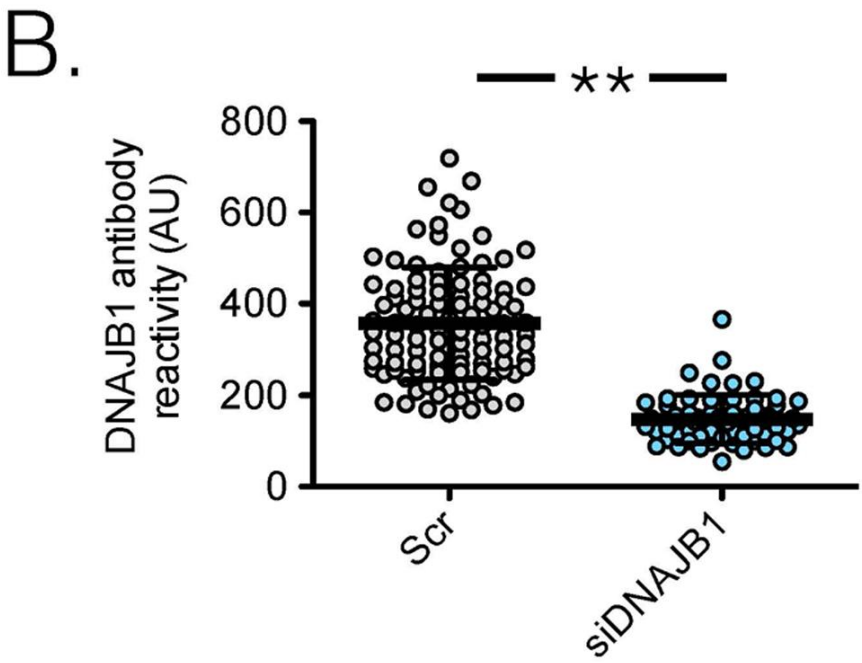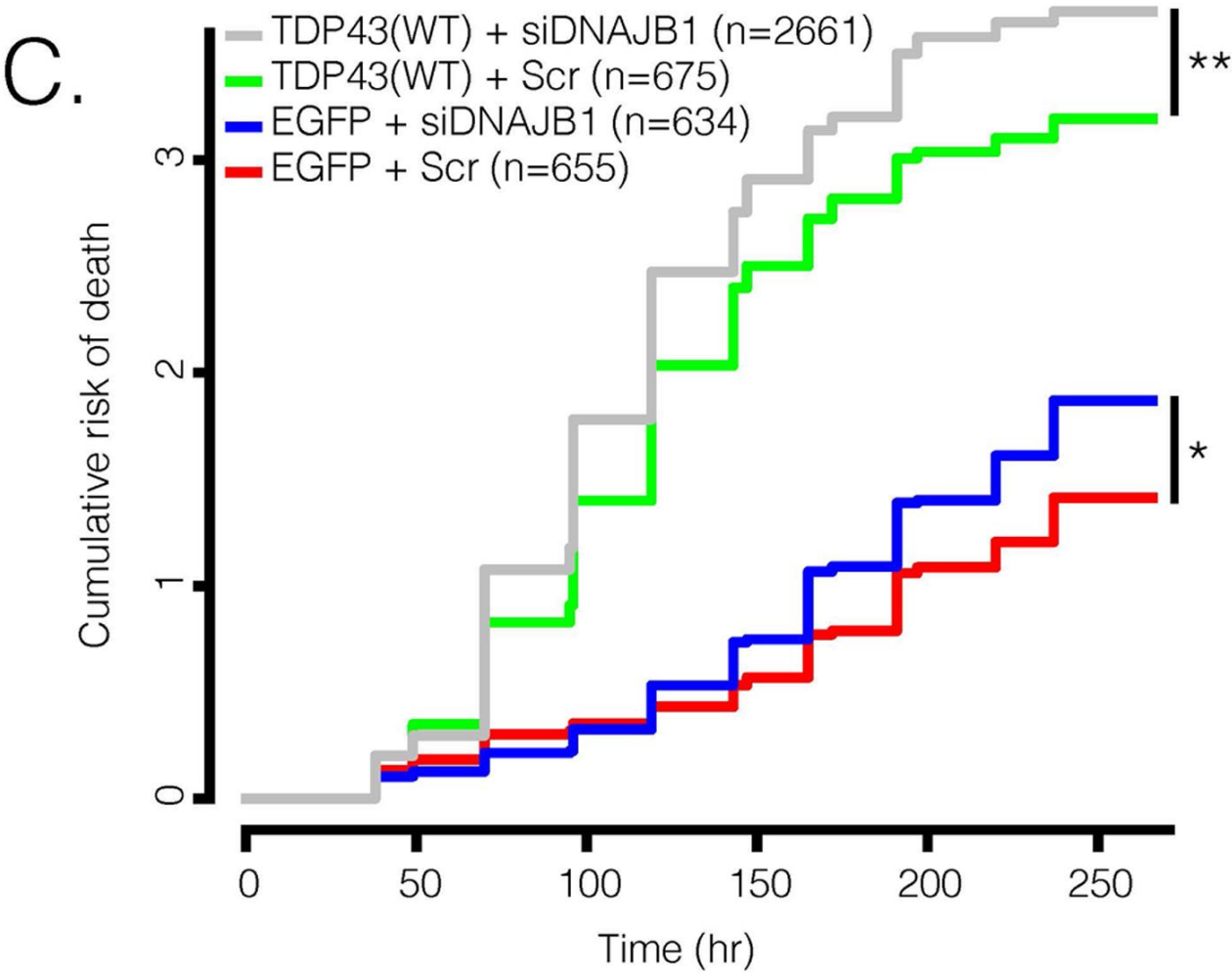

Supplement: S6 Fig — Rodent primary cortical neurons were dissected and transfected with plasmids encoding EGFP and TDP-43(WT)-mApple or mApple. In each case, neurons were also transfected with scrambled siRNA or siRNA targeting DNAJB1. (A) Knockdown was validated by immunocytochemistry using antibodies against DNAJB1. Scale bar, 50 μm. (B) Transfection with siRNA against DNAJB1 resulted in a 60% reduction in anti-DNAJB1 antibody reactivity (N = 101 and 62 neurons from Scr and siDNAJB1, respectively. ** p < 0.0001 by the Mann—Whitney U test. (C) In longitudinal assays of neuronal survival, DNAJB1 knockdown enhanced the risk of death by 20% in control neurons expressing EGFP alone and in neurons overexpressing TDP43. * HR 1.20, p 0.004; ** HR 1.21, p 2.3x10^-5^; # HR 3.18, p < 2x10^-16^, Cox proportional hazards analysis. Results were pooled from two independent experiments.* (PDF) [file pgen.1006805.s006.pdf]
