# Supplementary material for: Overexpression of the essential Sis1 chaperone reduces TDP-43 effects on toxicity and proteolysis
Source: PLoS Genet. 2017 May 22;13(5):e1006805. doi: 10.1371/journal.pgen.1006805 (PMC5460882; doi:10.1371/journal.pgen.1006805)

S2A Fig

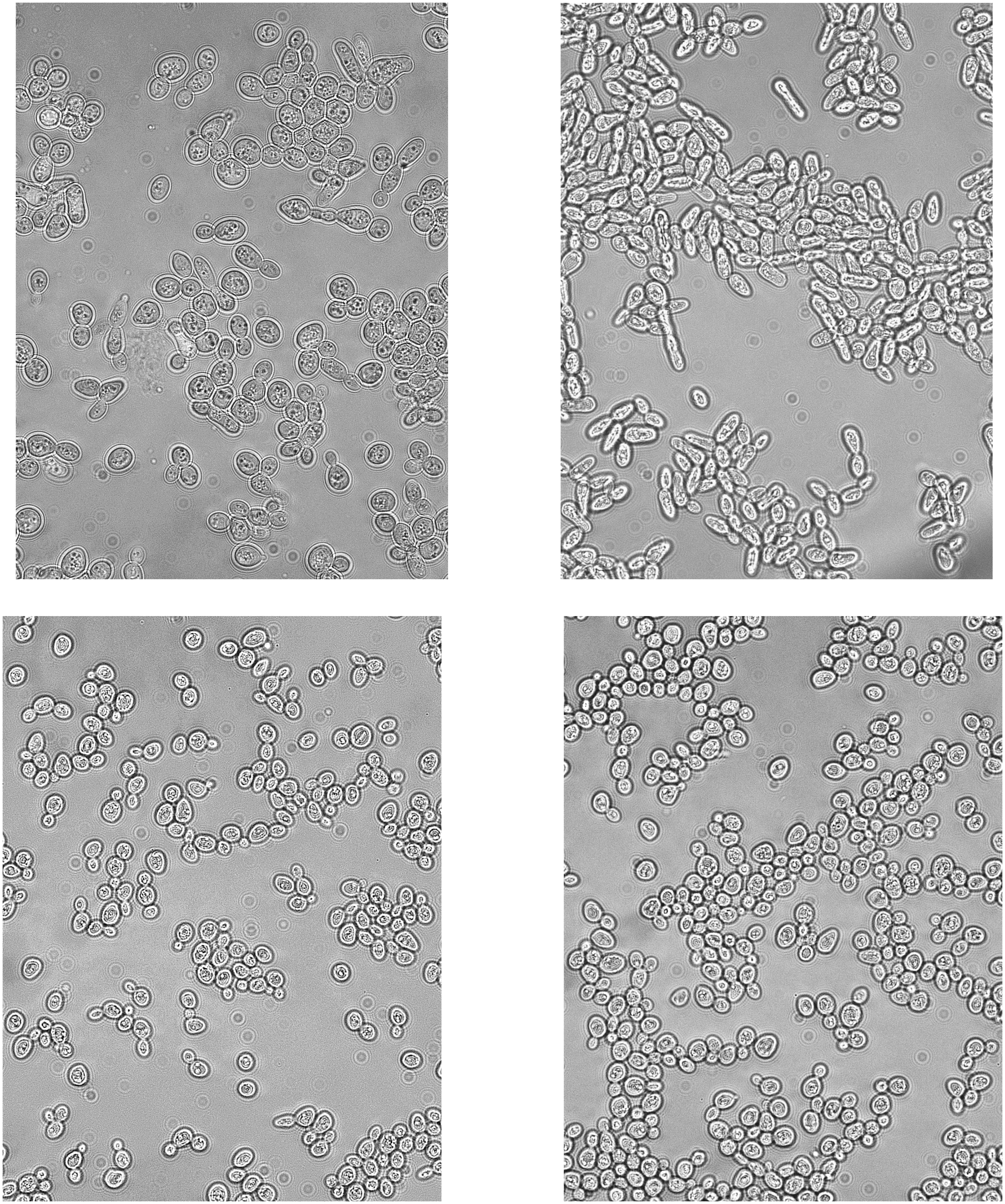

S2B Fig

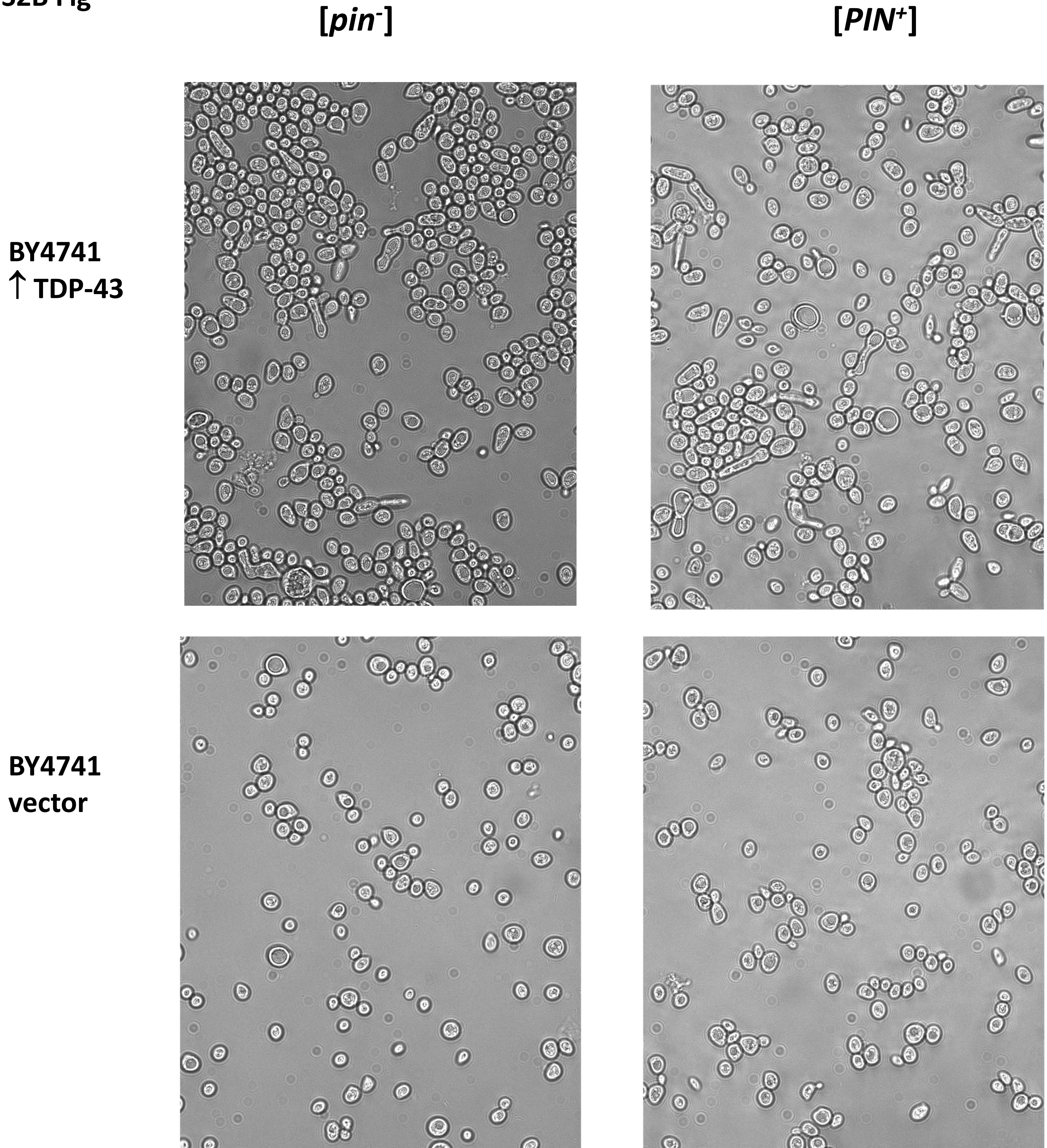

S2C Fig

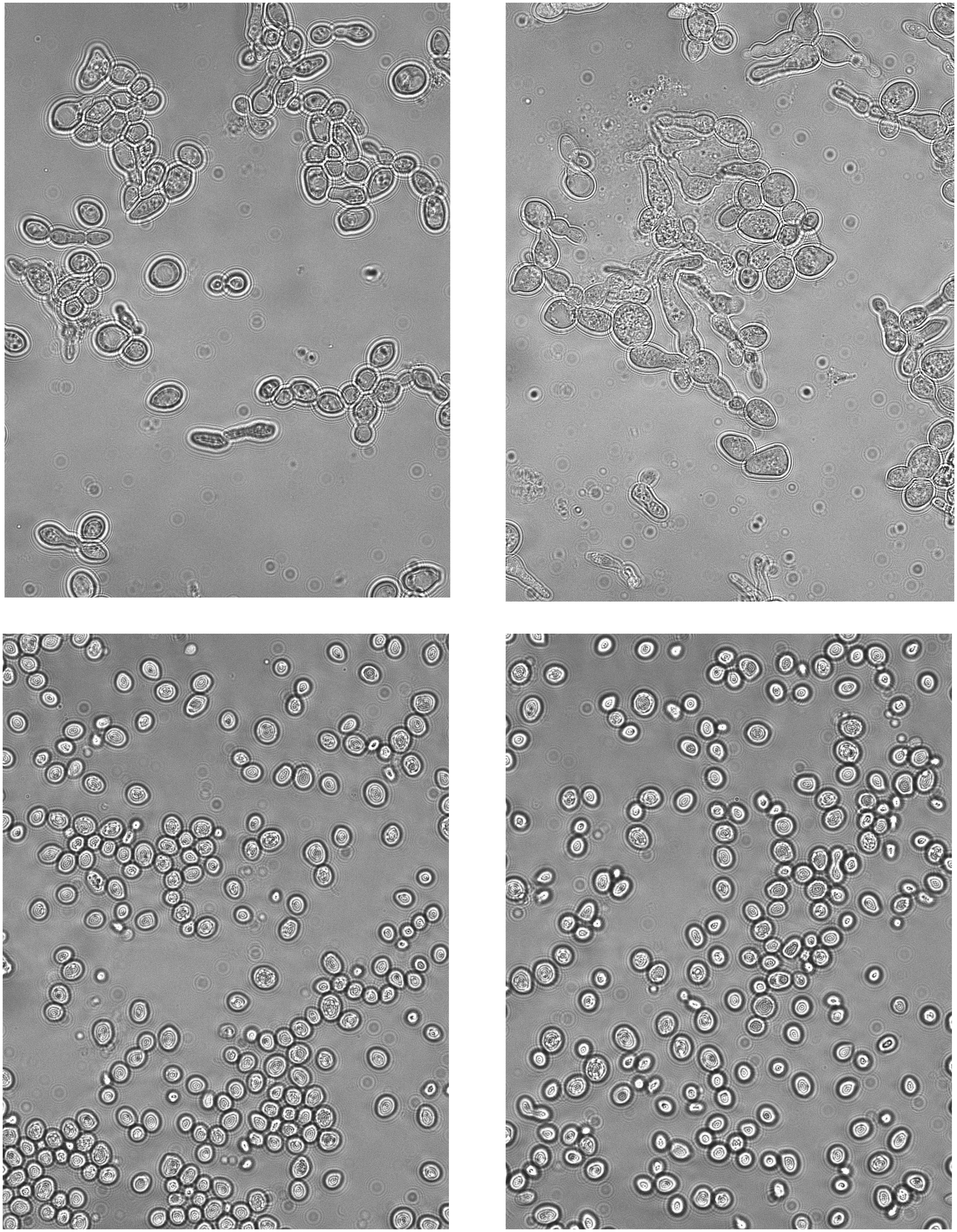

Supplement: S2 Fig — [PIN+] and [pin-] versions of 74D-694 (A), BY4741 (B) and W303 (C) carrying pGAL1-TDP-43-DsRed (p2173) or pAG415 GAL1-ccdB-DsRed (p2302) were grown on 2% galactose plates for 4 days and then imaged at the same magnification. Shown are representative fields of many fields photographed and examined. (PDF) [file pgen.1006805.s002.pdf]
